# Epigenetic-based combinatorial resveratrol and pterostilbene alters DNA damage response by affecting SIRT1 and DNMT enzyme expression, including SIRT1-dependent γ-H2AX and telomerase regulation in triple-negative breast cancer

**DOI:** 10.1186/s12885-015-1693-z

**Published:** 2015-10-12

**Authors:** Rishabh Kala, Harsh N. Shah, Samantha L. Martin, Trygve O. Tollefsbol

**Affiliations:** 1 Department of Biology, University of Alabama at Birmingham, 1300 University Boulevard, Birmingham, AL 35294 USA; 2 Comprehensive Center for Healthy Aging, University of Alabama at Birmingham, 1530 3rd Avenue South, Birmingham, AL 35294 USA; 3 Comprehensive Cancer Center, University of Alabama at Birmingham, 1802 6th Avenue South, Birmingham, AL 35294 USA; 4 Nutrition Obesity Research Center, University of Alabama at Birmingham, 1675 University Boulevard, Birmingham, AL 35294 USA; 5 Comprehensive Diabetes Center, University of Alabama at Birmingham, 1825 University Boulevard, Birmingham, AL 35294 USA

**Keywords:** Combination, Epigenetics, Apoptosis, Genomic stability, SIRT1, γ-H2AX, DNMTs, Telomerase, Breast cancer cells

## Abstract

**Background:**

Nutrition is believed to be a primary contributor in regulating gene expression by affecting epigenetic pathways such as DNA methylation and histone modification. Resveratrol and pterostilbene are phytoalexins produced by plants as part of their defense system. These two bioactive compounds when used alone have been shown to alter genetic and epigenetic profiles of tumor cells, but the concentrations employed in various studies often far exceed physiologically achievable doses. Triple-negative breast cancer (TNBC) is an often fatal condition that may be prevented or treated through novel dietary-based approaches.

**Methods:**

HCC1806 and MDA-MB-157 breast cancer cells were used as TNBC cell lines in this study. MCF10A cells were used as control breast epithelial cells to determine the safety of this dietary regimen. CompuSyn software was used to determine the combination index (CI) for drug combinations.

**Results:**

Combinatorial resveratrol and pterostilbene administered at close to physiologically relevant doses resulted in synergistic (CI <1) growth inhibition of TNBCs. SIRT1, a type III histone deacetylase (HDAC), was down-regulated in response to this combinatorial treatment. We further explored the effects of this novel combinatorial approach on DNA damage response by monitoring γ-H2AX and telomerase expression. With combination of these two compounds there was a significant decrease in these two proteins which might further resulted in significant growth inhibition, apoptosis and cell cycle arrest in HCC1806 and MDA-MB-157 breast cancer cells, while there was no significant effect on cellular viability, colony forming potential, morphology or apoptosis in control MCF10A breast epithelial cells. *SIRT1* knockdown reproduced the effects of combinatorial resveratrol and pterostilbene-induced SIRT1 down-regulation through inhibition of both telomerase activity and γ-H2AX expression in HCC1806 breast cancer cells. As a part of the repair mechanisms and role of SIRT1 in recruiting DNMTs, the effects of this combination treatment was also explored on DNA methyltransferases (DNMTs) expression. Interestingly, the compounds resulted in a significant down-regulation of DNMT enzymes with no significant effects on DNMT enzyme expression in MCF10A control cells.

**Conclusion:**

Collectively, these results provide new insights into the epigenetic mechanisms of a novel combinatorial nutrient control strategy that exhibits synergy and may contribute to future recalcitrant TNBC prevention and/or therapy.

**Electronic supplementary material:**

The online version of this article (doi:10.1186/s12885-015-1693-z) contains supplementary material, which is available to authorized users.

## Background

Breast cancer is among the most common cancers of women in the United States, second only to skin cancer. After lung cancer, it is the second leading cause of cancer death in women. Currently, an estimate of 231,840 new cases of invasive breast cancer are expected to be diagnosed and about 40,290 breast cancer deaths in women are expected in the year 2015 [1]. Out of the total breast cancers, around 10–20 % of breast cancer are designated as triple-negative breast cancers (TNBC). They lack potential biomarkers such as endocrine receptors: estrogen and progesterone receptors (ER and PR, respectively) and HER2 protein. They are typically associated with a poor prognosis and are recalcitrant to conventional hormonal therapies, in contrast to triple-positive breast cancers and HER2-positive breast cancers. Epidemiological studies have indicated that the incidence rate of cancers is higher in affluent nations where the diet and the environment contribute to genomic instabilities [2, 3]. In many developing countries, such as those in Asia where fruits and vegetables are the staple diet, incidences of cancer are remarkably lower [4]. Cancer has been viewed as a set of diseases that are driven by progressive genetic abnormalities as well as epigenetic alterations [5].

Diet-or environment-induced aberrations of genes may develop over time but are often reversible, indicating that only gene expression has changed while the actual DNA sequence remains unchanged. Such reversible gene-based alterations are mediated by a process referred to as epigenetics. Epigenetic changes are believed to occur at a higher frequency than sequence-based genetic changes. The common epigenetic modifications that occur are DNA methylation, histone modifications such as acetylation, methylation and phosphorylation, and chromatin remodeling. DNA methylation and histone modification play important roles in the transcriptional regulation of genes involved in cell cycle progression, proliferation, apoptosis, and cell death. Alterations in the epigenetic-controlled expression of these genes may mediate carcinogenic processes as well. This paper will focus on these two key players in regulating the epigenetic machinery and will further address down-stream effects of SIRT1 (Type III HDAC) down-regulation [6–8].

Recent studies have indicated that bioactive dietary components are strong epigenetic modulators that appear to play a part in prevention of breast cancer [8–10]. A growing group of studies has indicated that nutritional factors play an important role in many human diseases and have been identified and evaluated for their activities against cancer [11, 12]. One such food source is berries, which are rich sources of a wide variety of antioxidant phytochemicals such as stilbenes. Stilbenes are natural phenolic compounds, consisting of resveratrol and pterostilbene. Resveratrol (3, 4′, 5 trihydroxystilbene) is a natural phytoalexin, synthesized by plants such as grapevines, berries and peanuts in response to an injury [12].

Resveratrol is also enriched in the skin of red grapes, mulberries, peanuts and pines. The biological role of resveratrol is to protect plants against fungal infections (phytoalexin), especially against infection with *Botrytis cinerea*. Pterostilbene (3,5-dimethoxy-4-hydroxystilbene), found in blueberries and grapes, is also used as a part of Ayurvedic medicine for centuries. Chemically, pterostilbene is a dimethyl ether derivative of resveratrol and, like resveratrol, it is a phytoalexin.

Both of these key polyphenols have been speculated to mimic caloric restriction at the molecular level [12] and are proposed to affect DNA methyltransferase (DNMT) enzyme activity [13, 14]. However, for the first time we are reporting the combinatorial effects of these two compounds on inhibiting the activity of SIRT1, a type III histone deacetylase (HDAC). HDACs play a major role in various cellular processes by preventing chromatin accessibility [11]. Recent studies have shown the importance of HDAC inhibition and its possible implication in cancer therapy [8, 10, 11, 15].

SIRT1 is a mammalian homologue of the yeast silent information regulatory Sir2, which requires nicotinamide adenine dinucleotide (NAD+) as a cofactor for its action. It is a class III HDAC, which is responsible for modifying histones as well as some non-histone proteins through deacetylation and thereby regulating cell growth, apoptosis, stress response, adaptation to calorie restriction, metabolism, cellular senescence and tumorigenesis [16–18]. Recent studies have highlighted a unique feature of SIRT1 in regulating DNA damage and repair as well as its role in maintaining telomere length and genomic stability [11, 19, 20]. Moreover, previous studies have shown that SIRT1 deficiency impairs the formation of repair foci which can result in DNA damage, thereby protecting against tumors by inducting apoptosis [21, 22]. Interestingly, epigenetic patterns can be reversed, and epigenetic processes have in recent years become candidates for drug-mediated therapies.

In addition to the genetic changes caused by DNA damage, several tumors often contain epigenetically silenced protective genes with aberrant promoter region CpG island DNA hypermethylation [11]. Errors in double stranded break (DSB) repair can also cause mutations and chromosome instability that lead to cancer instead of cell death. SIRT1, when localized to the promoter of a gene, can also induce gene silencing by increasing the methylation of the promoter region of that gene by possibly engaging DNMTs to the damage site [21, 23]. Previous studies have shown involvement of SIRT1 in upregulating DNMT1 and DNMT3B presence, specifically at the DNA damage site [23]. Interestingly, SIRT1 has been found to localize at the promoters of these methylated-silenced tumor suppressor genes in some cancer cells, but not to promoters of the same tumor suppressor genes were they are normally in an active state [24].

In normal cells, SIRT1 has been linked to the role of protecting cells against potential carcinogenic agents and environmental stress. Whereas in malignant growth, it provides a strong stimulus which involves aberrant methylation as well as deacetylation of the promoter region of various protective genes and contributes to gene silencing and initiation and/or maintenance of cancer [18, 21, 23]. This could also be one of the potential mechanisms for overproduction of the telomerase enzyme, which enables cancer cells to replicate indefinitely. For the first time, we are reporting a relationship between SIRT1 and the human telomerase reverse transcriptase gene (*hTERT)* in human TNBC cells, thus opening a new area which requires further investigation. These findings suggest that DNA damage may directly contribute to the large number of epigenetically silenced genes in tumors due in part from hypermethylation [25–27] and histone deacetylation [10, 15] across the damage region [28] . *Human telomerase reverse transcriptase* (*hTERT*) encodes the catalytic subunit of telomerase and is an epigenetically-regulated gene. *hTERT* is over-expressed in more than ~90 % of human cancers but not in normal somatic cells. In recent years, *hTERT* has emerged as a promising target for cancer therapeutics and is an important marker for the diagnosis of malignancy [10, 29]. We have found that combinatorial resveratrol and pterostilbene resulted in down-regulation of *hTERT* at both the gene and enzymatic activity level.

The present study was undertaken to evaluate the combinatorial effects of resveratrol and pterostilbene treatment on TNBC cells. Understanding how these two dietary compounds work may provide important clinical implications for disease prevention and therapy, further aiding in the development of drugs that provide some of the health benefits of this dietary regimen. The goal of this study was to determine an optimal bioactive dietary compound combination regimen, which in turn may enhance future *in vivo* analyses and elucidate the translational chemopreventive potential of targeting epigenetic modulators involved in TNBC genesis.

## Results

### Combinatorial resveratrol and pterostilbene can synergistically inhibit the viability of TNBC cells with no significant effects on control MCF10A breast epithelial cells

**Fig. 1:**
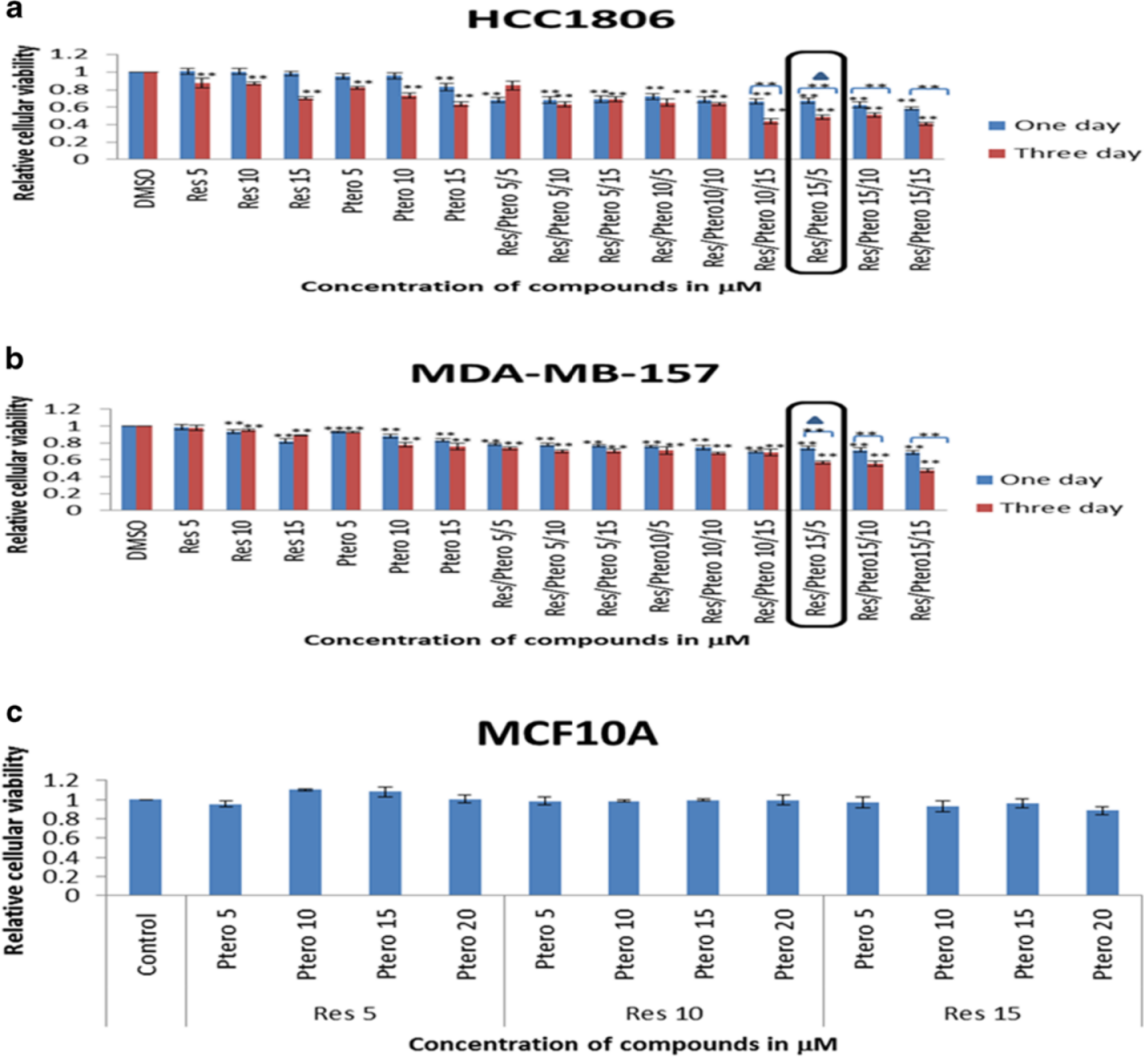
Time- and dose-dependency of resveratrol (Res) and pterostilbene (Ptero) in inhibiting cellular viability. Different concentrations of resveratrol and pterostilbene were used singly as well as in combination for 24 h (one day) and 72 h (three day) to determine time- and dose- dependent inhibition in both the breast cancer cell lines. With 72 h treatments, the combinatorial resveratrol and pterostilbene showed a highly significant inhibition in comparison to the DMSO control, both single and combinatorial treatments at 24 h and single doses at 72 h. After determining the significance, the combination index (CI) of these two drugs at 72 h was determined using CompuSyn software. Resveratrol at 15 μM and pterostilbene at 5 μM in combination were found to exhibit synergism (indicated by triangle) with the lowest CI value among all of the combinations used (See Additional files 1 and 2). **a** Effects of resveratrol and pterostilbene single and combinatorial treatments on HCC1806 breast cancer cells. With 72 h of combinatorial treatment, these cells incurred a highly significant inhibition in comparison to the DMSO control and different treatments at 24 h. **b** Effects of resveratrol and pterostilbene single and combination treatments on MDA-MB-157 breast cancer cells. This cell line also showed a highly significant inhibition at 72 h (three days) treatments in comparison to the DMSO and different treatments at 24 h. Both the cancer cell lines showed time- and dose-dependent inhibition and combinatorial 15 μM of resveratrol and 5 μM of pterostilbene was also found to exhibit synergism as determined by CI values. **c** Different concentrations of resveratrol and pterostilbene were used to treat MCF10A control cell breast cells for 72 h to determine toxicity. No significant inhibition was observed in comparison to vehicle control. Values are representative of three independent experiments and are shown relative to control ± SE; *P <0.05, **P <0.01. Res, resveratrol; Ptero, pterostilbene

**Fig. 2:**
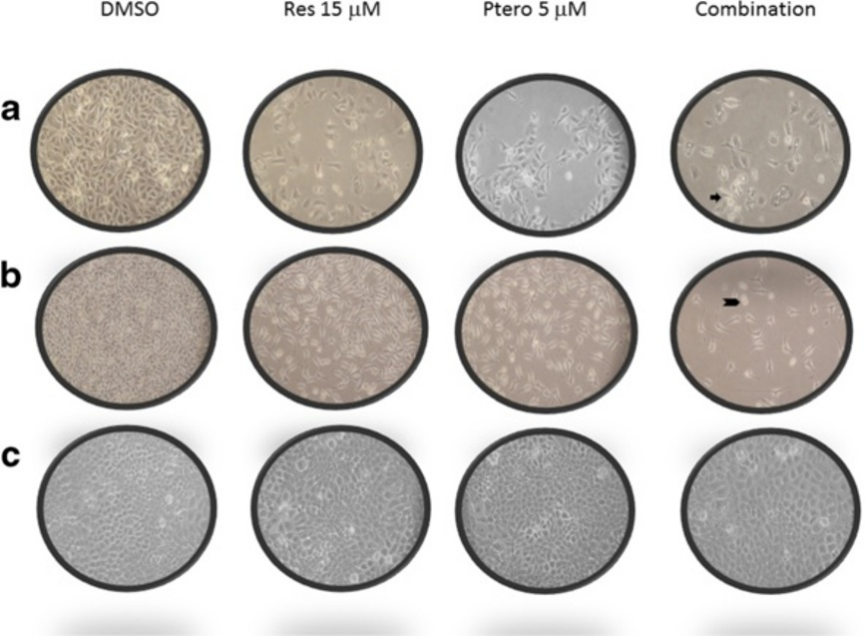
Morphology of breast cancer cell after 72 h of treatment in 6-well plates. **a** Effects of resveratrol and pterostilbene on HCC1806 breast cancer cells. With combinatorial treatments, cells showed changes in morphology with increased amorphous floating bodies indicating cell death (indicated by arrow). **b** Effects of resveratrol and pterostilbene on MDA-MB-157 cells. With combinatorial treatment, cells showed a decrease in cellular proliferation and became amorphous (indicated by chevron). **c** No change in cellular morphology was observed with single or combinatorial treatments of compounds on MCF10A breast epithelial control cells. Images were taken at 100X magnification

To determine the most effective concentration of these two dietary compounds on TNBC cells, MTT assays were performed. As shown in Fig. 1a and b, both the HCC1806 and MDA-MB-157 breast cancer cell lines showed time- and dose-dependency, with the most effective concentration of resveratrol at 15 μM and pterostilbene at 5 μM after 72 h treatments in comparison to individual treatments and DMSO control. The above combination did not show any significant effects on MCF10A control cells after 72 h of treatment as depicted in Fig. 1c. Furthermore, the addition of 15 μM of resveratrol and 5 μM of pterostilbene exhibited highly significant inhibitory effects when compared with single doses and combinatorial treatments at 24 h. This inhibitory effect of 15 μM of resveratrol and 5 μM of pterostilbene in combination was found to be synergistic (Combination index <1) in their mode of action in both TNBC cell lines as determined by CompuSyn software [30]. The morphology of human breast cancer cells treated with resveratrol and pterostilbene was also changed as shown in Fig. 2a and b. Combinatorial treatments resulted in more spherical cellular morphology with increased floating cells, indicating both cell death and inhibited cellular proliferation in these two breast cancer cell types. The equivalent doses of both resveratrol and pterostilbene alone as well as in combination were found to have no significant effects on control MCF10A breast cells (Fig. 2c) after 72 h of treatment, indicating safety at these levels. In order to investigate the long term effects of this combination on tested cell lines, colony forming assays were performed. The results are highlighted in Table 1 and the observations appear in Table 1 legend.

**Table 1:** Colony forming potential in TNBCs and MCF10A control cells

Treatments (10 days)	Cells plated	Colony counted	Plating efficiency (%)	Survival fraction (%)
M DA-MB-15 7				
DMSO	500	490	98	100.00
Res 15	500	399	79.8	81.43
Ptero 5	500	417	83.4	85.10
Combination	500	220	44	44.90
Treatments	Cells plated	Colony	Plating	Survival fraction
(14 days)		counted	efficiency	
MCF10A				
DMSO	500	165	32.96	100
Res 15	450	136	30.33	92.03
Ptero 5	500	175	35.06	106.3
Combination	480	145	30.13	91.44

After staining the tissue culture dishes (20 mm), colonies were counted using a colony counter. Large colonies consisting of 50 or more cells were counted. MDA-MB-157 breast cancer cells displayed a reduction in colony forming potential after different treatments as determined by survival fraction (%). This reduction was more effective in combination treatments when compared with single doses of resveratrol (15 μM) and pterostilbene (5 μM) alone. Interestingly, MCF10A control cells did not display a significant change in the colony forming potential when compared within the treatment groups and the DMSO treated group. These observations further provide the effectiveness of this combinatorial regimen in inhibiting breast cancer cells growth. However, HCC1806 breast cancer cells did not form any successful colonies in any of the groups (data not shown). Values are representative of three independent experiments

### Resveratrol and pterostilbene combination induces apoptosis in breast cancer cells with no significant apoptotic effects on MCF10A control breast cells

**Fig. 3:**
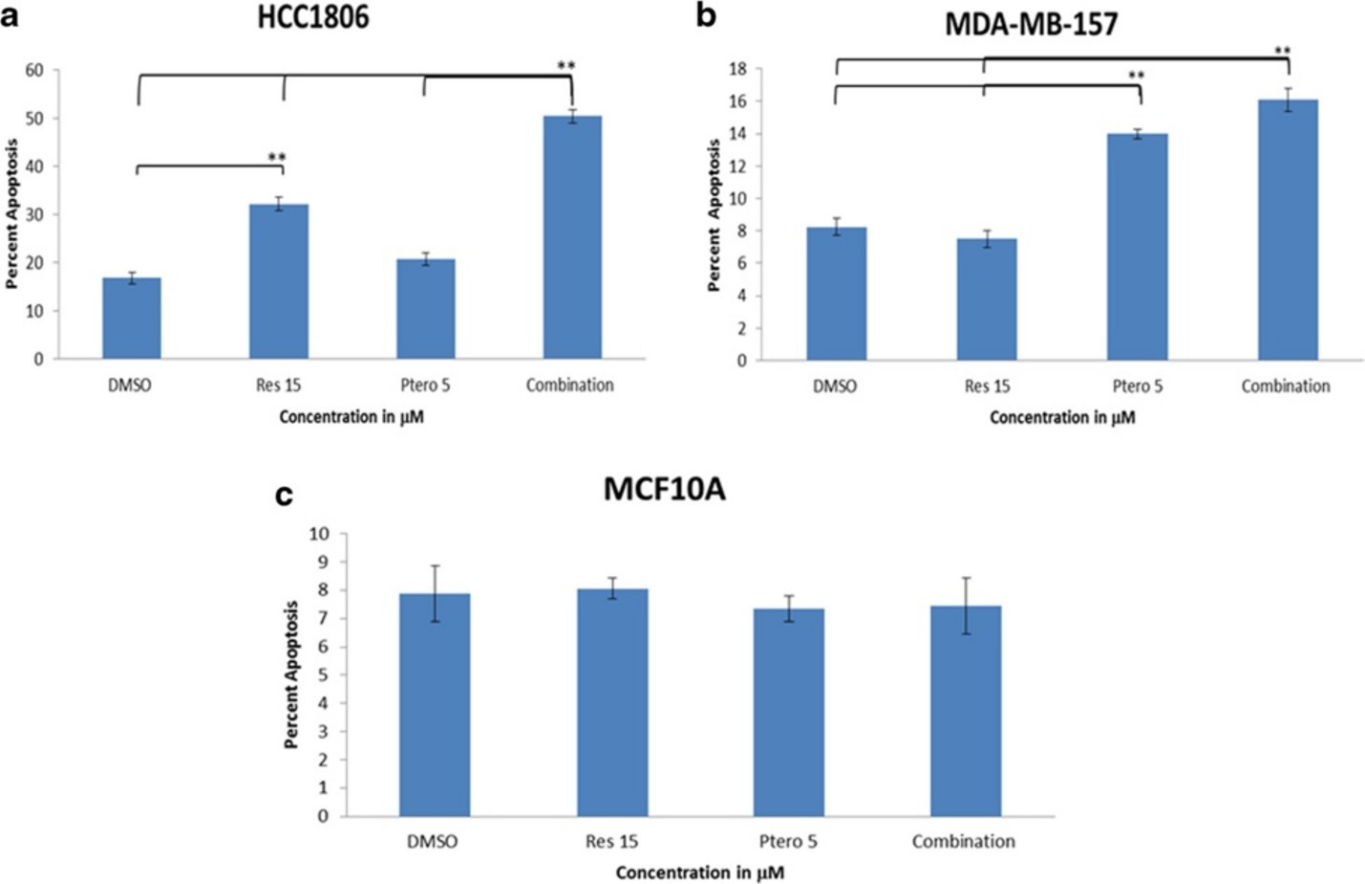
Combinatorial resveratrol and pterostilbene induces apoptosis in breast cancer cells. **a** HCC1806 cells undergo tremendous apoptosis after 72 h of treatment with resveratrol at 15 μM and combinational treatments at 15 μM resveratrol and 5 μM pterostilbene. Percent apoptosis in combinatorial treatment in HCC1806 cells was significantly elevated in comparison to DMSO (vehicle control) and individual treatments. **b** MDA-MB-157 cells underwent apoptosis with 5 μM pterostilbene and combinational treatment by 72 h. Both of the cell types were found to be highly significant in change in percent apoptosis in comparison to DMSO (vehicle control). **c** MCF10A cells did not show any significant change in apoptosis after 72 h of treatment with any of the tested compounds. Values are representative of three independent experiments and are shown as percent of control ± SE; *P <0.05, **P <0.01

A decrease in cell viability can be achieved by inhibiting cell cycle progression and/or by inducing cellular apoptosis. Induction of apoptosis is one of the major mechanisms by which a chemotherapeutic agent can be effective [8, 31]. To further determine the effectiveness of this combination regimen, we studied the apoptotic potential of resveratrol and pterostilbene on two different breast cancer cell lines. Figure 3a shows the effect of these two compounds on HCC1806 breast cancer cells. With 15 μM resveratrol and combinatorial treatment, there was a significant increase in apoptotic cells in relationship to DMSO (vehicle control). In addition, combinatorial treatment was also found to be highly significant with either 15 μM resveratrol or 15 μM pterostilbene alone. Figure 3b shows the effect of these two compounds on MDA-MB-157 breast cancer cells. Apoptosis was observed with both pterostilbene-treated (5 μM) and the combination set, which was highly significant in comparison to DMSO. In contrast to the above TNBC cell lines, there was no significant apoptosis observed in the MCF10A control breast cells (Fig. 3c) after 72 h of treatment with the respective compounds. These observations suggest that resveratrol at 15 μM and pterostilbene at 5 μM can induce cellular apoptosis without any effects on MCF10A control breast cells, thus suggesting their potential efficacy for future chemotherapy.

### Combined resveratrol and pterostilbene arrest HCC1806 cells predominantly in G2/M phase and MDA-MB-157 cells in both G2/M and S phase

**Fig. 4:**
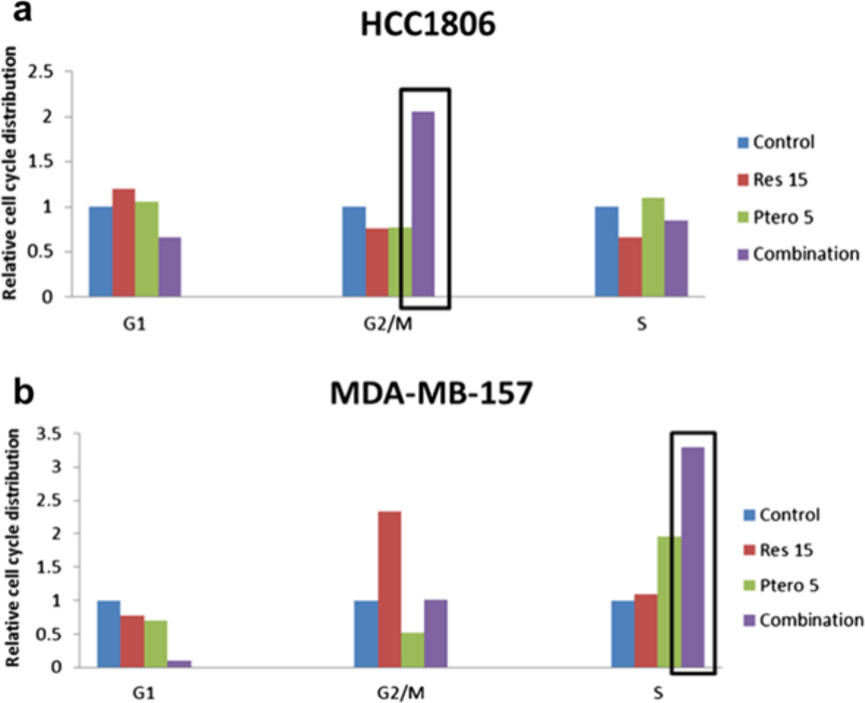
Resveratrol and pterostilbene arrest cells in G2/M and S phase of cell cycle. **a** HCC1806 breast cancer cells were arrested in G2/M phase with combinatorial treatment after 72 h. **b** MDA-MB-157 breast cancer cells were arrested in G2/M phase with 15 μM resveratrol and in S phase with combination treatment after 72 h. Data shown are representative of three different experiments

Cell cycle progression analysis revealed a predominant arrest of HCC1806 cells in G2/M phase (Fig. 4a), which could account for the significant level of apoptosis in these breast cancer cell lines upon combination treatment. Figure 4b depicts a shift of cells from G2/M phase with 15 μM resveratrol treatment to S phase with 5 μM pterostilbene and combination treatments.

### Combinatorial treatment inhibits SIRT1 in breast cancer cells

**Fig. 5:**
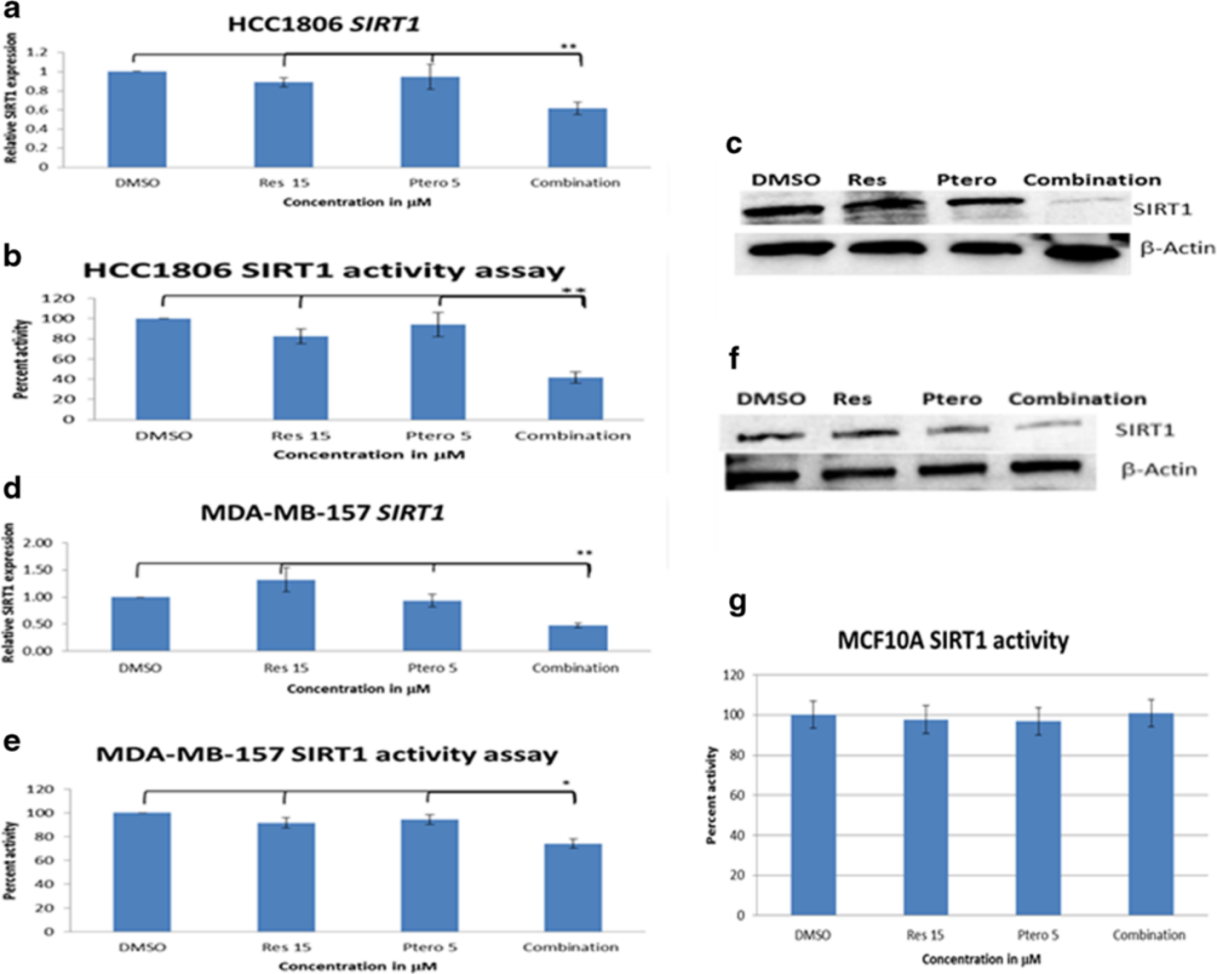
Combinatorial resveratrol and pterostilbene inhibited SIRT1 in TNBCs with no effects in breast control cells. **a** and **d** Relative real-time *SIRT1* mRNA expression after 72 h of treatments in HCC1806 cells and MDA-MB-157 breast cancer cell lines, respectively. With combination treatment, there was a significant down-regulation of *SIRT1* mRNA. GAPDH was used as the internal control. **b** and **e** Percent SIRT1 enzyme activity relative to DMSO control after 72 h of treatment with compounds alone as well as in combination in HCC1806 and MDA-MB-157 cells, respectively, using 20 μg of nuclear extract. With combinatorial treatment, a significant down-regulation in SIRT1 enzyme activity occurred which was found to be consistent with real-time data. **c** and **f** SIRT1 protein western blot after 72 h of treatment with compounds alone as well as in combination in HCC1806 and MDA-MB-157 cells, respectively. β-Actin was used as an internal control. With combination treatment, there was a decrease in SIRT1 protein as evident by western blot which is consistent with real-time and activity data. **g** No significant change observed in SIRT1 enzyme activity in MCF10A breast control cells after 72 h of treatment with compounds alone or in combination. Values are representative of three independent experiments and are shown as percent of control ± SE; *P <0.05, **P <0.01

To further understand the mechanism of action of resveratrol and pterostilbene, we investigated the effects of these compounds on SIRT1. This important enzyme is a class III histone deacetylase (HDAC), which is responsible for modifying histones as well as some non-histone proteins through deacetylation and thereby regulating cell growth, apoptosis, stress response, adaptation to calorie restriction, metabolism, cellular senescence and tumorigenesis. Figure 5 demonstrates the effects of resveratrol and pterostilbene on SIRT1 in HCC1806 and MDA-MB-157 breast cancer cells, respectively. Figure 5a and d show effects at the mRNA level and Fig. 5b and e show the effects of combination treatment on enzyme activity, represented as a percentage relative to DMSO. In both the cell lines, with an optimal combination of 15 μM resveratrol and 5 μM pterostilbene, there was a significant inhibition of SIRT1 at both the gene and enzyme level in comparison to single-compound administration and DMSO treatments. Figure 5c and f show the western blot analysis of SIRT1 in HCC1806 and MDA-MB-157 breast cancer cells and further confirm these inhibitory effects. Interestingly, there was no significant change in the relative percent activity of SIRT1 in MCF10A control breast cells (Fig. 5g).

### Resveratrol and pterostilbene alter DNA damage response (DDR) in breast cancer cell lines

**Fig. 6:**
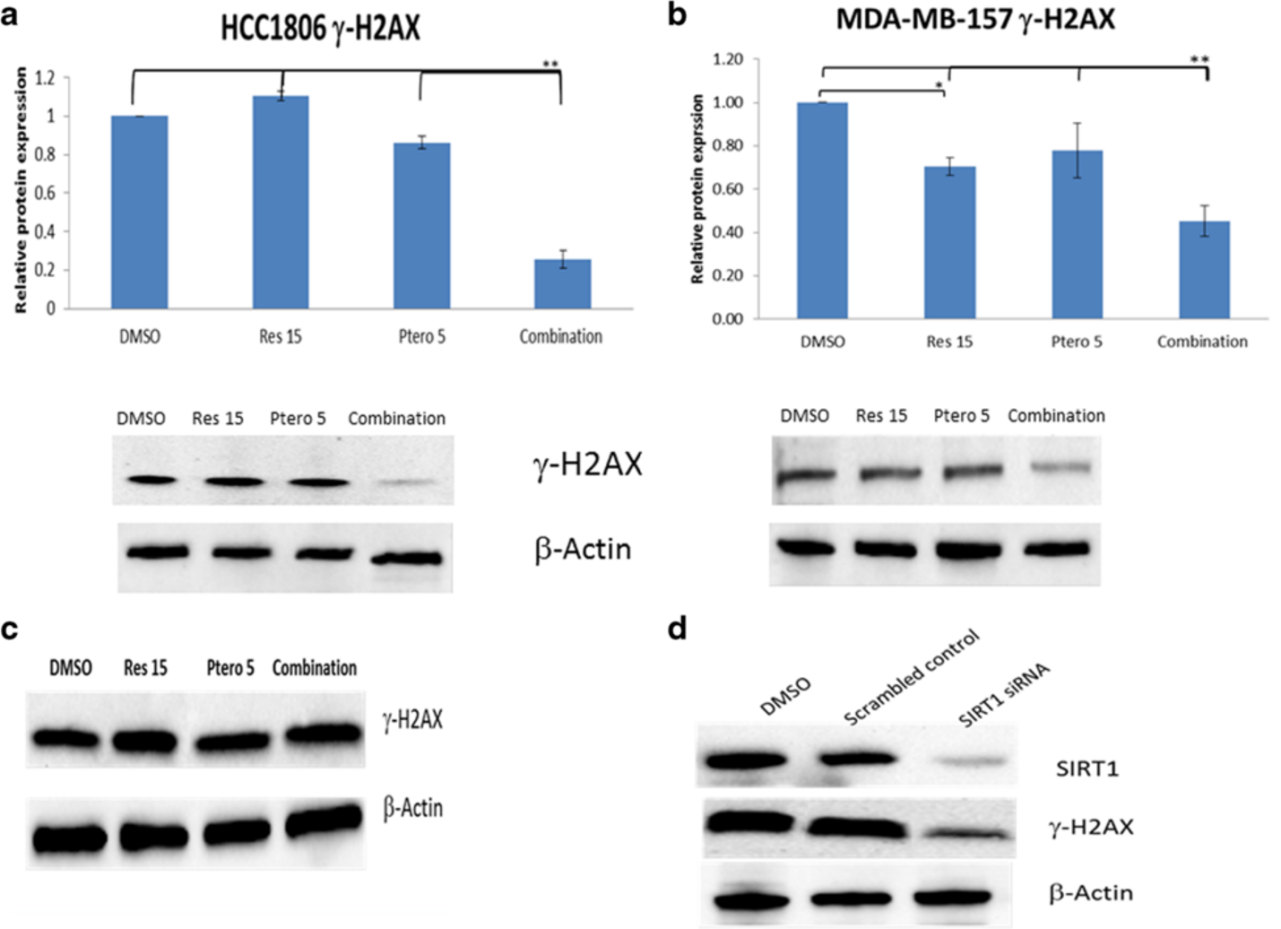
Resveratrol and pterostilbene effects on γ-H2AX expression. **a** Densitometry analysis and western blot of γ-H2AX protein after 72 h of treatment with compounds alone as well as in combination in HCC1806 breast cancer cells. β-Actin was used as an internal control. With combination, there was significant decrease in γ-H2AX protein expression. Values are representative of three independent experiments ± SE; *P <0.05, **P <0.01. **b** Densitometry analysis and western blot of γ-H2AX protein after 72 h of treatment with compounds alone as well as in combination in MDA-MB-157 breast cancer cells. β-Actin was used as an internal control. With combination, (*P* <0.01) as well as 15 μM resveratrol (*P* <0.05) treatment, there was a decrease in γ-H2AX protein expression, and combination treatment was found to be highly significant (*P* <0.01) when compared with all the treatment groups. Values are representative of three independent experiments ± SE; *P <0.05, **P <0.01. **c** Effect of compounds alone as well as in combination on g-H2AX protein in MCF10A control cells after 72 h of treatment. β-Actin was used as an internal control. No significant change in g-H2AX protein expression was observed with the following treatments. **d** SIRT1 knockdown western blot in HCC1806 cells using 3 μl of transfecting agent and 30 nM of *SIRT1* siRNA. With the knockdown, there was a decrease in γ-H2AX protein expression. Scrambled control did not show any effects. β-Actin was used as an internal control. Data shown are representative of three separate experiments

**Fig. 7:**
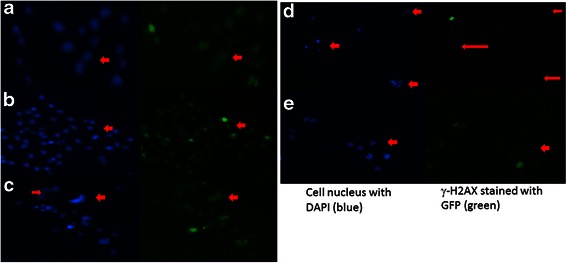
Effects of combination and SIRT1 knockdown on γ-H2AX protein expression in HCC1806 breast cancer cell line as analyzed by immunofluorescence microscopy. DAPI was used to stain cell nuclei, shown in blue color. Alexa Fluor®488 (GFP) shown in green was used to stain the target protein. **a** Shows the expression of γ-H2AX protein in green with live cells stained in blue after 72 h of DMSO treatment in HCC1806 breast cancer cells. **b** and **c** The effect of 15 μM resveratrol and 5μM pterostilbene treatments after 72 h are demonstrated. Images show no change in the intensity of blue and green color between the two sets as marked by the red arrows. **d** Effects of combination after 72 h of treatment depict a significant change in the intensity of green color (γ-H2AX protein) in comparison to the blue color stained nuclei. **e** Effect of *SIRT1* knockdown in HCC1806 breast cancer cells. This resulted in decreased γ-H2AX protein expression (green color) as marked by red arrows

Research has shown that chromatin structure plays a critical role in DDR following DNA damage. Phospho-H2AX is a marker for the DNA damage and repair mechanism. Various cancers are marked by unchecked cell cycles which usually results in increased rates of DNA damage and mutation, causing an increased expression of γ-H2AX [32]. In recent years, SIRT1 has been reported to regulate the DNA damage and repair mechanism along with H2AX [21]. In the present study, the combination treatment of 15 μM resveratrol and 5 μM pterostilbene resulted in apoptosis in both the breast cancer cell lines. To further understand the mechanism of action of these two compounds, phopho-H2AX levels were analyzed using western bolting (Fig. 6) and immunofluorescence (IF) microscopy (Fig. 7). Figure 6a reveals a highly significant (*P* <0.01) decrease in γ-H2AX in HCC1806 cells subjected to combinatorial treatment. MDA-MB-157 cells also displayed a decrease in γ-H2AX level with both 15 μM resveratrol and combination treatment (Fig. 6b). Effects of compounds were also analyzed in MCF10A control cells (Fig. 6c). Importantly, there was no change in the expression of γ-H2AX as analyzed by western blot in the control cells. To further strengthen these findings and determine the mechanism of action of these two compounds, SIRT1 knockdown was performed and its effects observed on γ-H2AX in HCC1806 cells. Figure 6d shows a decrease in the expression of γ-H2AX, further providing a link between SIRT1 and γ-H2AX. In order to confirm this decrease in γ-H2AX levels, IF microscopy was performed in HCC1806 breast cancer cells (Fig. 7). SIRT1 knockdown was also performed to establish a connecting link between SIRT1 down-regulation and genomic instability by targeting γ-H2AX protein. DAPI (blue) was used to stain cell nuclei and Alexa Fluor®488 GFP (green) was used to stain γ-H2AX protein. It was interesting to observe a decrease in γ-H2AX protein in combination treatments as well as in SIRT1 knockdown set in HCC1806 breast cancer cells as depicted in Fig. 7. These observations were found to be consistent with our previous western blot findings (Fig. 6).

### Effects of the combination regimen on DNA methyltransferase enzymes (DNMTs) in HCC1806 breast cancer cells and MCF10A control cells

**Fig. 8:**
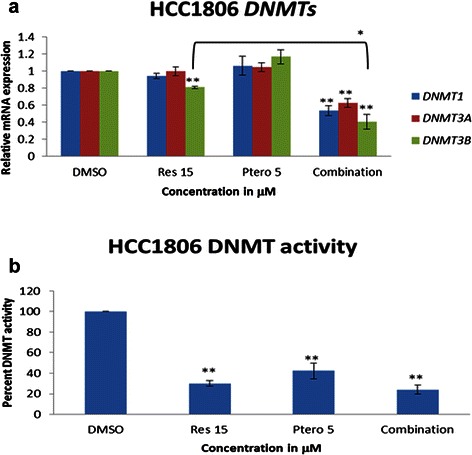
Combination treatments altered epigenetic enzyme expression and their activity in HCC1806 breast cancer cells. **a** Resveratrol and pterostilbene after 72 h of treatment affected *DNMTs* (*DNMT1, DNMT3A, DNMT3B*) mRNA expression. With the combination of resveratrol and pterostilbene all three DNMTs were significantly down-regulated. Resveratrol at 15 μM also resulted in a decrease in *DNMT3B* mRNA expression after 72 h. *GAPDH* was used as an internal control. **b** Overall DNMT enzyme activity analysis, using 20 μg of nuclear extract. After 72 h, the entire treated group resulted in a significant down-regulation of enzyme activity in comparison to DMSO control. There was no significant difference in overall enzyme activity within the treatment group itself. Values are representative of three independent experiments and represented as percent of control ± SE; *P <0.05, **P <0.01

**Fig. 9:**
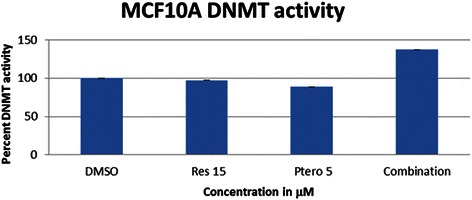
Combination treatments did not alter epigenetic enzyme expression and their activity in MCF10A control cells. Overall DNMT enzyme activity analysis, using 20 μg of nuclear extract. After 72 h, there was no significant change in the enzyme activity in comparison to DMSO control in MCF10A control cells further highlighting the effectiveness of this combination regimen. Values are representative of three independent experiments and represented as percent of control ± SE; *P <0.05, **P <0.01

To further explore the mechanism for our observations, we next focused on HCC1806 cells since few differences were observed between the HCC1806 and MDA-MB-157 cell types in the prior studies. To help elucidate the mechanism of action of these two polyphenols, their effects were observed on DNMTs expression. Figure 8a shows the effect of these compounds on the relative mRNA expression of *DNMT1*, *DNMT3A* and *DNMT3B* in HCC1806 breast cancer cells. With the combination treatment, all the DNMTs underwent a significant down-regulation at the mRNA level. Further, the effects of these polyphenols were observed on overall DNMT enzyme activity. Figure 8b shows a significant decrease in overall enzyme activity with resveratrol (15 μM), pterostilbene (5 μM) and combination treatment. To further strengthen this study, overall DNMT enzyme activity was analyzed in MCF10A control cells. Figure 9 shows no significant change in the overall enzyme activity within the treatment groups.

### Resveratrol and pterostilbene inhibits *hTERT* expression in HCC1806 cells

**Fig. 10:**
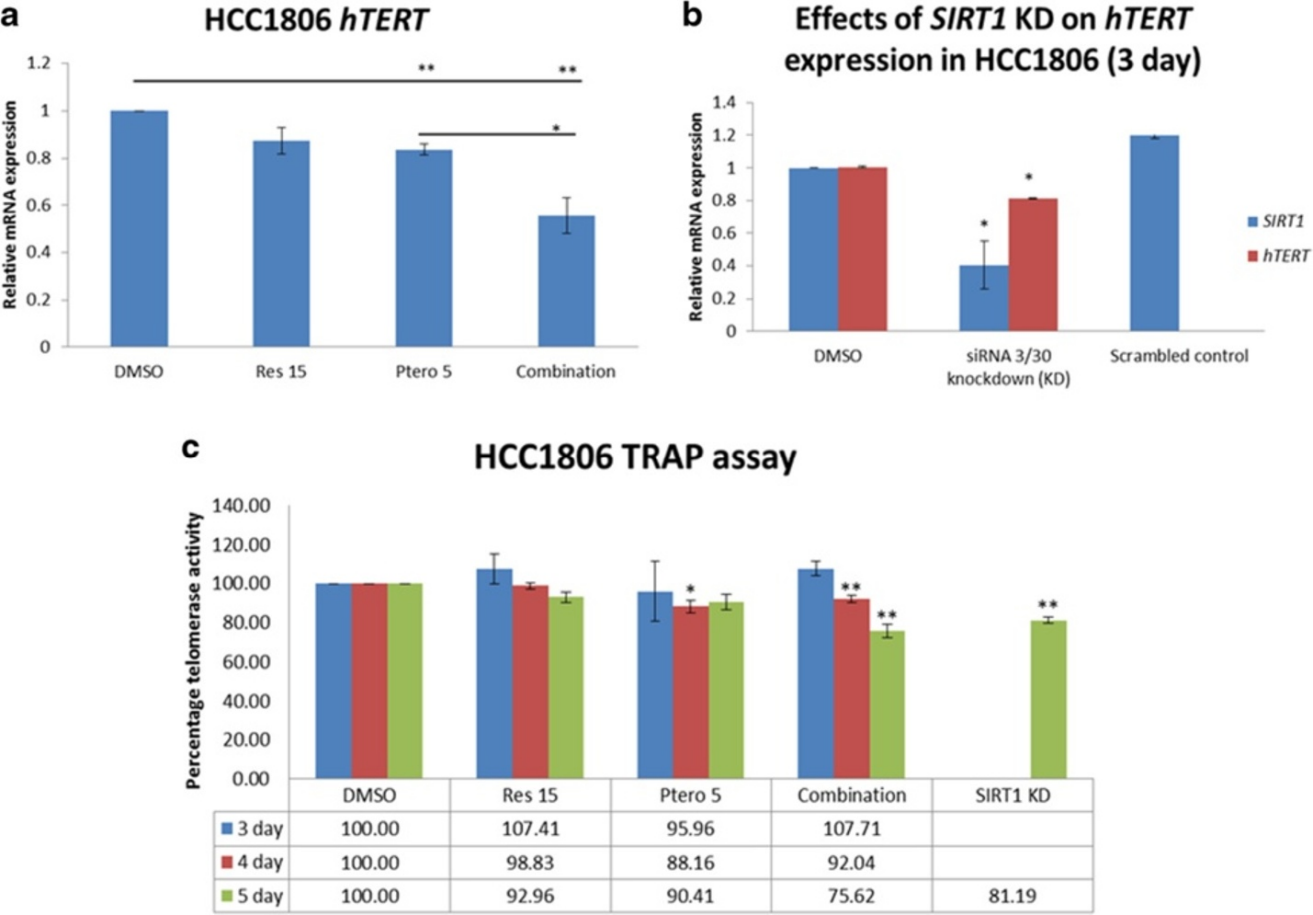
Effects of combination and *SIRT1* knockdown, on *hTERT* expression in HCC1806 breast cancer cells. **a** Combination (Res/Ptero) treatment after 72 h rendered a significant down-regulation of *hTERT* mRNA levels as shown by real-time PCR. GAPDH was used as an internal control. Values are representative of three independent experiments ± SE; *P <0.05, **P <0.01. **b** *SIRT1* knockdown (*SIRT1 KD*) was performed and its effects on *hTERT* mRNA were analyzed using real-time PCR. After 72 h of knockdown, there was a significant down-regulation of both *SIRT1* and *hTERT* mRNAs. No significant effects were observed with scrambled siRNA. GAPDH was used as an internal control. Values are representative of three independent experiments ± SE; *P <0.05, **P <0.01. **c** Telomerase enzyme activity was analyzed using TRAP assays in the HCC1806-treated and knockdown set. At the fourth and fifth days of treatment, there was a significant down-regulation of telomerase activity with the combination of resveratrol and pterostilbene. *SIRT1* knockdown also resulted in significant down-regulation of telomerase enzyme activity at fifth day. Values are representative of three independent experiments and are shown as percent of control ± SE; *P <0.05, **P <0.01

To explore the mechanistic effects of resveratrol and pterostilbene, the expression level of human telomerase reverse transcriptase (*hTERT)* was analyzed. The *hTERT* gene has previously been noted to be under epigenetic regulation [10, 31]. As shown in Fig. 10a, relative *hTERT* mRNA level is down-regulated after resveratrol and pterostilbene combination treatment. Furthermore, the telomerase activity assay also indicated an overall time-dependent decrease as analyzed by telomeric repeat amplification protocol (TRAP) assay and depicted in Fig. 10c.

### SIRT1 knockdown resulted in *hTERT* down-regulation in HCC1806 breast cancer cells

To further explore the mechanism of action of these two dietary polyphenols and to determine the role of SIRT1 in telomerase expression, knockdown of *SIRT1* was performed. As shown in Figs. 10b and 6d, a successful SIRT1 knockdown was performed as depicted by RT-PCR and western blot analysis. Figure 10b shows a decrease in *hTERT* mRNA level in the *SIRT1* knockdown set in comparison to the control. To further confirm the cross-talk between SIRT1 and hTERT, TRAP analysis was performed as shown in Fig. 10c. There was a significant decrease in telomerase activity in *SIRT1* knockdown sample, suggesting an existing cross-talk between these two key cancer-associated proteins.

### Effects of compounds on telomerase enzyme activity in MCF10A control cells

**Fig. 11:**
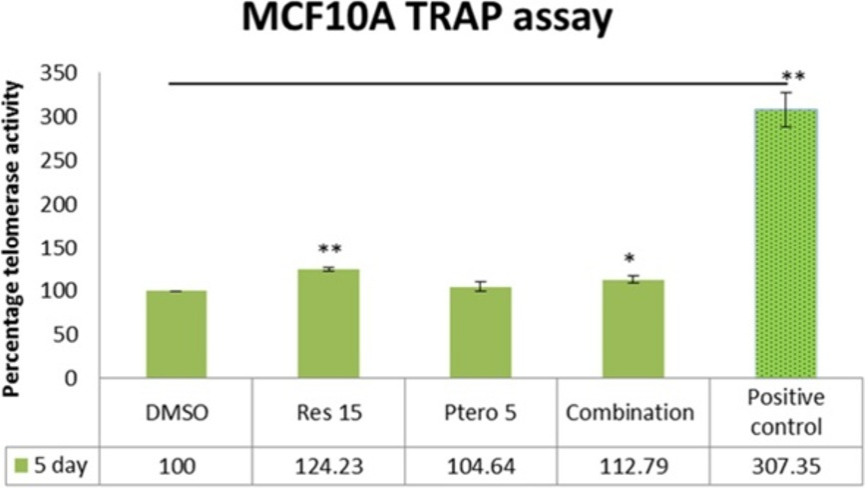
Effects of compounds on telomerase enzyme in MCF10A breast control cells. Telomerase enzyme activity was analyzed using TRAP assay in the MCF10A control cells. After five days of treatment, there was a significant up-regulation in telomerase activity in 15 μM resveratrol (*P* <0.01) treated group and combination treatment (*P* <0.05) when compared with the DMSO treatment. Pterostilbene (5 μM) did not show any significant change in the telomerase enzyme activity. A positive control (PC) was used in the experimental design in order to understand this up-regulation in the treated group. The difference in the enzyme activity between the treatment groups and PC was highly significant (*P* <0.01). Moreover, the PC displayed around 200 fold more active telomerase enzyme as compared to the experimental values. Values are representative of three independent experiments and are shown as percent of control ± SE; *P <0.05, **P <0.01

In order to further understand the effectiveness and fidelity of the compounds used in this study, we analyzed the expression of telomerase in MCF10A control cells using TRAP activity kit. From the previous results in this study which highlighted a time-dependency of the telomerase enzyme, MCF10A cells were treated with the compounds for 5 days and then analyzed. Figure 11 showed a slight increase in the telomerase enzyme activity at 15 μM resveratrol and combination treatments. However, this increase in the enzyme activity is insignificant when compared with a positive control, as shown in Fig. 11.

## Discussion

Most compounds used in chemotherapy are synthetic or analogs of compounds that are present in dietary food sources. The application of these compounds in treatment often requires high dosage and prolonged exposure for therapy-related effectiveness. Unfortunately, such treatments often result in problems such as non-specific tissue-cell cytotoxic effects and multi-drug resistance tumors. To avoid these issues, it is imperative that the chemicals used in the treatment should be safe, easily available and cost-effective. Most dietary epigenetic phytochemicals meet these criteria and can be administered as dietary supplements, thereby offering promising new options for the development of more effective chemopreventive and chemotherapeutic strategies [10]. Resveratrol and pterostilbene are two phytoalexins produced by plants in response to an infection. Ideally, they are the plants’ own defense system. Once the mechanistic pathways of phytochemicals are determined, modification of these compounds to increase their stability without compromising their therapeutic efficacy can be achieved [9]. The combination of two biomolecules can synergistically or additively improve the therapeutic impact on tumors at lower doses than when used alone.

In the present study, resveratrol or pterostilbene single treatment, as well as combination treatment, was administered to breast cancer and control breast cells. The concentrations we used in this study model are close to a normal physiologically achievable range [33, 34]. Depending on the expression of the receptors, breast cancers can be classified into various groups. Breast cancers which are positive for estrogen receptor (ER), progesterone receptor (PR), and human epidermal growth factor receptor 2 (HER2) represent discrete biological entities with distinct clinical profiles and are often associated with better prognosis and can be treated with hormone therapy. In contrast, women with triple-negative breast cancers [TNBC (i.e., ER-, PR-, and HER2-)] are typically associated with less favorable prognosis [35, 36] and account for about 10–20 % of breast cancers. Breast cancers which are ER-, PR- and HER2-negative cannot be successfully treated with hormonal therapies or medications that work by blocking the receptor and are often fatal. We undertook this study in part to address this important challenge. TNBC used in this study were MDA-MB-157 and HCC1806 cells. MCF10A breast non-tumorigenic epithelial cells were used as a control cell line to determine the toxicity and efficacy of our combinatorial dietary regimen.

Cell viability analysis was performed for 24 h and 72 h using various concentrations of resveratrol and pterostilbene to determine time- as well as dose-dependency in all breast cancer cell lines. The combination effect was also analyzed on breast control (MCF10A) cells for 72 h to detect any evidence of toxicity. Resveratrol at 15 μM with pterostilbene at 5 μM after 72 h was found to be the most effective combination and was highly significant when compared with DMSO and 24 h of combination treatments. This combination in both the cell lines was found to possess synergism when compared with different combination doses after 72 h of treatments. Combination index (CI) values at 72 h of treatment for HCC1806 and MDA-MB-157 breast cancer cells were found to be the lowest with respect to other CI values (See Additional files 1 and 2). The CompuSyn software used to calculate synergy generates different values of combination index where CI <1, synergism; CI = 1, additive effects; CI >1, antagonism, and have been employed for drug combination and general dose effect analysis in various studies [30, 37]. Our analysis using CompuSyn software indicated that combination treatments lower than 15 μM resveratrol and 5 μM pterostilbene at 72 h showed less effectiveness and qualitatively displayed a degree of antagonism, addictiveness and/or moderate synergism in both types of breast cancer cells (see Additional files 1 and 2). Collectively, both resveratrol and pterostilbene were found to exhibit dose- and time-dependent inhibitory effects on TNBC cells. Interestingly, the same concentration was used to treat breast control MCF10A cells and did not result in any significant inhibition. To further confirm the effects over a long term, colony forming assays were performed in the tested cell lines (Table 1). Depending on the growth rate and colony forming potential of the cells, MDA-MB-157 breast cancer cells were analyzed for 10 days and MCF10A control cells were analyzed for 14 days. The combination of two polyphenols displayed a decreased in the colony forming potential of MDA-MB-157 cells with no significant decrease in MCF10A control cell. Ironically, HCC1806 breast cancer cells did not form successful colonies (≥50 cells). Cells were plated at an increased seeding density from 200 cells/plate to 1000 cells/plate from 7 days to 15 days, none of them formed successful colonies (data not shown). One explanation for this uncertainly could be due to critical density phenomenon in which cells won’t grow if they are too widely dissociated and makes too few contacts with other cells. The goal of this study was to determine the effects of resveratrol and pterostilbene combination treatment on both of these TNBC cell lines and to reveal the potential mechanism responsible for the effect. Our results indicated for the first time that resveratrol and pterostilbene can synergistically inhibit cellular viability in combination and can further impact cellular proliferation through cell cycle arrest and apoptosis induction in TNBC cells. Interestingly, the combination treatment in HCC1806 cells resulted in a tremendous apoptosis induction in comparison to DMSO and individual treatments. Moreover, treatment of MDA-MB-157 cells also resulted in apoptosis although the rate of apoptosis in comparison to HCC1806 cells was less but nevertheless significant.

Progress through each phase of the cell cycle is regulated carefully to avoid proliferation. Cells can be arrested in G1, S and G2/M phases to prevent any replicative errors. Cancer cells are characterized by unchecked cell cycles, often resulting in uncontrolled replicative errors and DNA damage. Previous studies have shown the role of p53 in arresting cells in G1 phase but not in S and/or G2/M phases [31]. Our experiments with resveratrol and pterostilbene combination treatment in HCC1806 and MDA-MB-157 cells resulted in predominant G2/M phase and S phase arrest, respectively. These observations have been found to be consistent with previous studies involving defective p53 status in various cancer cell lines [8, 31]. Resveratrol and pterostilbene combination at close to physiologically achievable concentration are shown in this investigation to inhibit cancer cell growth by inhibiting cell cycle progression and by the induction of apoptosis in these TNBC cells, with no significant effects in MCF10A control cells.

SIRT1, a class III histone deacetylase (HDAC) has been linked in normal cells to a role in protecting cells against potential carcinogenic agents and environmental stress. Whereas, in malignant growth it provides a strong stimulus, involving aberrant methylation and deacetylation of the promoter region of various genes and contributes to gene silencing, resulting in initiation and/or maintenance of cancer [17–19, 25]. *SIRT1* knockdown also results in cell cycle arrest and apoptosis induction. SIRT1 is one of the key epigenetic-modifying enzymes which, by itself or in complex, implements various cellular events such as DNA damage response (DDR), regulating stress, stabilizing the damage DNA along with γ-H2AX and DNMT’s and stabilizing eroded telomeres [11, 18, 21, 28, 38, 39]. For the first time, we have reported a significant down-regulation of SIRT1 with this combinatorial dietary approach. This down-regulation was confirmed at the transcriptional and translational as well as enzymatic levels. There was no significant change observed at the enzyme activity in MCF10A breast epithelial control cells, further suggesting the effectiveness and utility of this combinatorial approach.

DNA double-strand breaks are serious lesions which can lead to genomic instability and to cancer. A key marker and a way to monitor the DNA damage and repair mechanism is Phospho-H2AX [38]. H2AX is a member of the histone H2A family, one of the five histones that form chromatin along with eukaryotic DNA. SIRT1, along with γH2AX and ataxia telangiectasia mutated (ATM), is recruited to the DNA damage site and participates in the formation of repair foci [18, 21] and plays a role in stabilizing repair foci containing DNA repair factors. This mark is maintained at the break site until the break is repaired [18, 21]. Consistent with previous studies, cell lines with defective p53 show a longer persistence of γH2AX, as an indicator of stress [32]. Resveratrol and pterostilbene in combination showed a significant down-regulation of γH2AX (72 h) in both tested cancer cell lines with no effects on MCF10A control cells, further demonstrating the effectiveness of this dietary regimen in affecting DDR. This could also account for the increased apoptosis in both the cancer cell lines with this combination treatment. This γH2AX down-regulation in HCC1806 breast cancer cells was also confirmed using IF microscopy and interestingly was found to be consistent with our western blot data.

Recent studies demonstrated the recruitment of DNA methyltransferases (DNMTs) enzymes along with SIRT1 to the site of DNA damage as a part of the repair mechanism, which might be defective in cancer cells, resulting in hypermethylation of CpG-island promoters across the double stranded break [17, 23]. DNA methyltransferases and histone modifying enzymes are no longer considered independent epigenetic regulators, but are supposed to work in tandem or in cohesion to alter the epigenome. HDACs and DNMTs have been shown to assist with the DNA damage response by recruiting various other repair proteins to the site of damage. HDAC inhibitors such as butyrate and trichostatin A (TSA) have been shown to affect the repair machinery [18]. Double-stranded breaks can cause the development of cancer and also result in the induction of a hypermethylated state with the aid of silencing factor, SIRT1. Thus, in cancer cells we may observe increased expression of SIRT1, DNMTs and γ-H2AX proteins, as they are the key markers for genomic stress, DNA damage and repair foci formation [18, 23, 32, 38]. These two dietary polyphenols when administered (72 h) in HCC1806 breast cancer cells resulted in down-regulation of *DNMT1*, *3A* and *3B* at the gene level as well as at the enzymatic activity level in HCC1806 breast cancer cells. Interestingly, there was no significant change in overall DNMT enzyme activity in MCF10A control cells after 72 h of treatment. Thus, the observed molecular and cellular effects in this investigation could be due to a combined effect of this dietary regimen on SIRT1 and DNMT enzymes levels.

With every cell division eroded-critically short telomeres reveal double-strand breaks resulting in cellular senescence in normal mammalian cells [40], whereas under abnormal circumstances, they transform into cancer cells in lieu of senescence and/or apoptosis. The catalytic subunit of the enzyme telomerase (hTERT) is highly expressed in ~90 % of human cancers [10, 31]. Previous studies with normal human umbilical cord fibroblasts and stem cells have reported the role of SIRT1 in increasing the transcription of *hTERT* either directly by the involvement of FOXO3a and/or indirectly by affecting c-MYC [20, 39]. For the first time, we have reported down-regulation of *hTERT* with the use of resveratrol and pterostilbene in combination at close to physiological relevant doses in HCC1806 breast cancer cells. This down-regulation was evidenced by both real-time PCR and telomerase activity (TRAP) assay. Consistent with the previous studies, a time-dependent inhibition of telomerase enzyme was observed because of the stable half-life of telomerase enzyme [41, 42]. To further understand the mechanism of *hTERT* down-regulation, *SIRT1* knockdown was performed in HCC1806 breast cancer cells. It was discovered that *SIRT1* knockdown in human breast cancer cells resulted in a decrease in *hTERT* mRNA expression at the third day and enzyme activity at the fifth day of treatment as evidenced by RT-PCR and TRAP activity, respectively. One explanation for this hTERT down-regulation, which could account for growth inhibition in the breast cancer cells, could be due to a decrease in the downstream target of SIRT1, FOXO3a and c-MYC, which has been shown to increase *hTERT* expression in a SIRT1-dependent manner [20, 39]. Alternatively, many proteins involved in DDR also play a key role in telomere maintenance. One such protein is SIRT1, along with γ-H2AX and DNMTs, which are documented to maintain and stabilize the repair site and are significantly down-regulated in the present study [11, 28, 39]. Overall the effect of this dietary regimen was also analyzed on MCF10A control cells in order to determine the effectiveness of this approach. At the fifth day of the treatment there was a slight increase in telomerase activity in resveratrol (15 μM) and combination treatments when compared with the DMSO set, but this increase in activity was significantly below the positive control TRAP readings.

In the future, this dietary regimen may be used to provide a safe and effective treatment for TNBCs. It is documented that targeting γ-H2AX may also enhance the cytotoxic effects of irradiation therapy (IR), while circumventing adverse effects on unirradiated cells [43]. This down-regulation is also documented to sensitize cells to DNA damaging agent such as cisplatin [44]. Hence, combining DNA damaging agents (such as IR and cisplatin) with the current dietary regimen might provide a better treatment option. SIRT1, a key silencing factor known to be involved in a variety of cellular processes including DDR response and γ-H2AX expression, is shown in our study to participate in regulating *hTERT* expression, thereby opening future investigation which includes promoter-specific analysis of *hTERT* gene.

## Conclusion

Our results indicate that combinatorial resveratrol and pterostilbene at close to physiological-relevant concentration, synergistically and significantly inhibit proliferation of both MDA-MB-157 and HCC1806 breast cancer cells with no significant effects on control MCF10A breast epithelial cells, providing effectiveness and safety of this dietary regimen. This combination treatment resulted in predominantly G2/M and S phase cell cycle arrest along with apoptosis induction in these two TNBC cell lines. For the first time, this dietary regimen demonstrated down-regulation of type III HDAC, SIRT1, and impacted the DNA damage and response mechanism, further reflected by down-regulation of γ-H2AX and *hTERT* expression and increased apoptosis. HDAC inhibition appears to facilitate cancer cell death and holds a potential promise in the development of future cancer therapies. Plant-based dietary polyphenols used in this study targeted DNMT’s activity, which might further affect some methylation patterns downstream and is subject for future studies. Results obtained so far indicate the unique role of these two polyphenols in affecting breast cancer and opens new possibilities and pathways to target in cancer. This combination can also be used to study the effects on miRNA, which hold a great potential in regulating epigenetics events in the cells.

## Methods

### Cell lines

Breast cancer cell lines MDA-MB-157, HCC1806 and MCF10A were obtained from ATCC. MDA-MB-157 and HCC1806 are both triple-negative (ER-, PR-, HER2-) and p53 null. Clinically, MDA-MB-157 cells were initially obtained from a 44 year-old black female with medullary carcinoma of breast and HCC1806 cells were obtained from 60 year-old black female with acantholytic squamous carcinoma of the breast. This allowed us to target two broad age groups of aggressive breast cancers with triple-negative status. MCF10A is an immortalized, non-tumorigenic epithelial cell line used as a control in this study as commonly done [10]. MDA-MB-157 cells were grown in DMEM media (Mediatech Inc, Manassas, VA) and HCC1806 cells were grown in RPMI 1640 media (Mediatech Inc, Manassas, VA) supplemented with 10 % fetal bovine serum (Atlanta Biologicals, GA) and 1 % penicillin/streptomycin (Mediatech). MCF10A cells were grown in F12-DMEM media supplemented with the necessary antibiotics and growth supplements as required by the ATCC protocol. The cells were subcultured at 75 % confluence. All of the cell lines were maintained in an incubator at 5 % CO_2_ with a controlled temperature of 37 °C.

### Chemicals

Resveratrol (>99 % pure; GC) and pterostilbene (>97 % pure; HPLC) were purchased from Sigma-Aldrich. The compounds were prepared in dimethyl sulfoxide (DMSO), which was obtained from Sigma-Aldrich and stored at a stock concentration of 50 mM at –20 °C. Cells were treated with fresh resveratrol (Res) and pterostilbene (Ptero) every 24 h (one day) for up to 72 h (three days) after seeding. DMSO (1 μL/1 ml) was used as the vehicle control.

### MTT analysis

The number of viable cells in each well was estimated by the uptake of the tetrazolium-salt, 3-(4, 5-dimethylthiazol-2-yl)-diphenyltetrazoliu bromide (MTT). Approximately 5000 breast cancer cells were plated in 96- well plates and allowed to attach to the bottom of the plate overnight. Cells were then treated with increasing doses of resveratrol (5, 10, 15 μM) or pterostilbene (5, 10, 15 μM) or combinatorial resveratrol and pterostilbene (5 + 5, 5 + 10, 5 + 15, 10 + 5, 10 + 10, 10 + 15, 15 + 5, 15 + 10, 15 + 15 μM) for one day and/or three days to determine dose-as well as time-dependency. At the end of each treatment, cells were incubated with 100 μ1 of 1 μg/ml MTT for 2 h at 37 °C. The converted purple insoluble formazan, by mitochondrial enzyme, was further dissolved using 100 μl of DMSO. Readings were acquired at 595 nm using a microplate reader (iMark™, Bio-Rad). Relative cell viability was calculated in comparison to DMSO vehicle control.

### Morphological analysis

After determination of the optimal and safe concentrations of dietary compounds, approximately 8 × 10^4^ cells/2 ml were plated in 6-well plates. Medium containing freshly added resveratrol (15 μM), pterostilbene (5 μM) and combination of resveratrol + pterostilbene (15+5 μM) was added for 72 h. Morphology of cells was observed under a phase contrast microscope at magnification of 100×. Images of cells were captured with a Nikon Coolpix P5100 (Nikon, Tokyo, Japan).

### Colony forming assay

Cell were plated overnight in 6-well plates and then treated the next day for up to 24 h. Following treatment, single cell suspensions were obtained. The number of cells in each sample were counted carefully using a hemocytometer and diluted such that appropriate cell numbers are seeded into tissue culture dishes. Dishes were arranged in an incubator at 5 % CO_2_ with a controlled temperature of 37 °C. The incubation time for colony formation varies from 1 to 3 weeks for different cell lines. After incubation, colonies were fixed using 100 % ice-cold methanol for 10–15 mins. After fixing, colonies were stained using 0.1 % CV for 10–15 min and then washed with distilled water and dried overnight. Colonies were counted using a colony counter and plating efficiency and survival fraction were calculated as follows: Plating efficiency (PE) = (number of colonies counted/number of cells plated)*100 and Survival fraction (SF) = (PE of treated sample/PE of control)*100.

### Apoptosis analysis

Induction of apoptosis in human breast cancer cells caused by resveratrol and pterostilbene treatment alone or in combination was quantitatively determined by flow cytometry using Annexin V-conjugated Alexafluro 488 (Alexa488) Apoptosis Vybrant Assay Kit. Approximately 8 × 10^4^ cells/2 ml were plated in 6-well plates. Medium containing freshly added resveratrol (15 μM), pterostilbene (5 μM) and combination of resveratrol + pterostilbene (15+5 μM, respectively) was added for 72 h. Following treatment, cells were collected from 6-well plates by trypsinization, washed with PBS, and incubated with Alexa488 and propidium iodide (PI) for cellular staining in Annexin-binding buffer at room temperature for 10 min in the dark. The stained cells were analyzed by fluorescence-activated cell sorting (FACS) by using a FACS-Caliber instrument (BD Biosciences) equipped with Cell Quest 3.3 software.

### Cell cycle analysis

Flow cytometric assays were performed to assess the effects of resveratrol and pterostilbene alone or in combination on breast cancer cells. Approximately 8 × 10^4^ cells/2 ml were plated in 6-well plates. Medium containing freshly added resveratrol (15 μM), pterostilbene (5 μM) and combination of resveratrol + pterostilbene (15+5 μM, respectively) was added for 72 h. After treatment, cells were fixed using 70 % ethanol overnight and then were washed, pelleted and re-suspended in 0.04 mg/ml of PI, 0.1 % TritonX-100 and 100 mg/mL RNase in PBS. Stained DNA contents were analyzed with flow cytometry.

### Reverse transcription-polymerase chain reaction

RT-PCR was used to examine the expression of genes of interest. RNA was extracted using RNeasy Kit (Qiagen, Valencia, CA). One μg was reverse transcribed to cDNA using a cDNA synthesis kit (Bio-Rad).

### Real-time PCR

To determine the quantitative expression of genes of interest, Real-time PCR was performed. After 72 h of treatment with single as well as combination of two dietary bioactive compounds, cells were harvested, RNA was extracted and cDNA was prepared as described above. Primers (Table 2) were obtained from Integrated DNA Technologies, Inc. All the reactions were performed in triplicate and SYBR green Supermix (Bio-Rad) was used as fluorescent dye in Roche Light cycler 480. Thermal cycling was initiated at 94 °C for 4 min followed by 35 cycles of PCR (94 °C, 15 s; 60 °C, 30 s; 72 °C, 30 s). GAPDH was used as an endogenous control and vehicle control was used as a calibrator. The relative changes in gene expression were calculated using the following formula: Fold change in gene expression, 2^-ΔΔCt^ = 2^-{ΔCt (treated samples)-ΔCt (untreated control^ ^samples)}^, where ΔCt = Ct (genes of interest) – Ct (GAPDH) and Ct represents threshold cycle number.

**Table 2:** Primer sequences, forward and reverse, used for realtime PCR analysis

Gene	Forward primer	Reverse primer
GAPDH	5′-ACC ACA GTC CAT GCC ATC AC-3′	5′-TCC ACC CTG TTG CTG TA-3′
SIRT1	5′-TGG CAA AGG AGC AGA TTA GTA GG-3′	5′-CTG CCA CAA GAA CTA GAG GAT AAG A-3′
DNMT1	5′-TAC CTG GAC GAC CCT GAC CTC-3′	5′-CGT TGG CAT CAA AGA TGG ACA-3′
DNMT3A	5′-TAT TGA TGA GCG CAC AAG AGA GC-3′	5′-GGG TGT TCC AGG GTA ACA TTG AGS′
DNMT3B	5′-GGC AAG TTC TCC GAG GTC TCT G-3′	5′-TGG TAC ATG GCT TTT CGA TAG GAS′
hTERT	5′-AGG GGC AAG TCC TAC GTC CAG T-3′	5′-CAC CAA CAA GAA ATC CAC C-3′

### Western blotting

Protein extractions were performed by RIPA Lysis Buffer (Upstate Biotechnology, Charlottesville, VA) according to the manufacture’s protocol. Protein concentration was further determined with the Bradford method of protein quantification using the Bio-Rad Protein Assay (Bio-Rad; Hercules, CA). About 50 μg of the whole cell protein extract were loaded onto a 4–15 % Tris–HCl gel (Bio-Rad) and separated by electrophoresis at 150 V until the dye front ran off the gel. Separated proteins were then transferred to a nitrocellulose membrane using Trans-Blot Turbo transfer system (Bio-Rad) at 25 V for 7 min. After successful transfer, membranes were blocked in 0.5 % dry milk in Tris Buffered saline solution with 1 % Tween (TBST) using SNAP i.d. protein detection system. Primary and secondary antibody incubation was carried out according to the manufacturer’s protocol. Membranes were probed with the following monoclonal antibodies: SIRT1 (ABcam), Phospho H2AX (Cell signaling) and β-actin (Cell signaling). Immunoreactive bands were visualized using the Bio-Rad ChemiDoc XRS^+^ system.

### Immunofluorescence (IF) microscopy

Cells were treated and plated for 72 h in small petri plates (Corning®). After successful treatments, cells were prepared for IF microscopy following cell signaling technology protocol. Primary monoclonal antibodies were used for Phospho-H2AX (Cell signaling). Anti-Rabbit IgG Alexa Fluor®488 conjugate (Cell Signaling) green were used as secondary antibody. DAPI-blue (VECTASHIELD) was used as antifade mounting medium. Prepared samples were observed using NIKON AZ100M microscope (in dark).

### SIRT activity assay

Cultured breast cancer cells were harvested at the indicated time points and nuclear extract was prepared using nuclear extraction reagent (Pierce, Rockford, IL). The activity of SIRT was performed according to the manufacturer’s protocol using Epigenase Universal SIRT activity/Inhibition assay kit (EpigentecK, Brooklyn, NY).

### DNMTs activity assay

DNA methyltransferases (DNMTs) are a family of enzymes that transfer a methyl group to DNA. Cultured breast cancer cells were harvested at the indicated time points and nuclear extract was prepared with the nuclear extraction reagent (Pierce, Rockford, IL). Overall DNMT activity was determined using the EpiQuik DNA Methyltransferase Activity Assay Kit (Epigentek) according to the manufacture’s protocol. This analysis provides the levels of overall DNMT activity and is not specific to any particular gene or to any particular DNMT, and data are represented in terms of percentage control.

### Small interfering RNA (siRNA) knockdown

HCC1806 breast cancer cells were grown in 6-well plates and allowed to incubate overnight. The *SIRT1* siRNA was obtained from Santa Cruz Biotechnology. After dilution with nuclease free water, 30 nM siRNA was delivered to the cells using 3 μL of Silencer siRNA Transfection agent (Ambion/Applied Biosystems, TX, USA) according to the manufacturer’s instructions. Scrambled non-targeting siRNA (Santa Cruz Biotechnology) was used as a negative control to determine off-targets. After 72 h of knockdown, cells were harvested and checked for knockdown using RT-PCR and western blot.

### Telomerase activity assay (TRAP)

Telomerase activity was measured using TeloTAGGG telomerase PCR ELISA kit (Roche applied science, Indianapolis, IN) according to the manufacturer’s protocol. Total protein (5 μg) was added to the reaction mixture, and the generated telomere product was PCR amplified using 30 cycles (25 °C for 20 min, 94 °C for 5 min, 94 °C for 30 sec, 50 °C for 30 sec, 72 °C for 10 min). The amplified PCR product (5 μL) was subjected to treatment for the ELISA assay. The color change was measured within 30 min at 450 nm and with 690 nm as the reference wavelength, using an ELISA plate reader (Bio-Rad). Relative activity was measured in comparison to the DMSO control.

### Quantification of combination index

In order to determine the effects of combination of these two compounds on breast cancer cells, CompuSyn software was used. This software generates combination index (CI) values which further assists determination of the nature of the combination in comparison to single compound effects. It is freely available at http://www.combosyn.com/feature.html. CI <1, CI = 1, CI >1 represent synergism, additivity, or antagonism, respectively.

### Statistical analysis

All the data were determined from at least three independent experiments. Statistical significance of differences between the values of treated samples and control was determined by one way ANOVA using GraphPad Prism version 4.00 for Windows, graphPad Software (www.graphpad.com). In each case, *p* <0.05 and *p* <0.01 was considered statistically significant and highly significant, respectively.

## Additional files

Additional file 1: **Is the report generated after using CompuSyn software to calculate the synergism in HCC1806 breast cancer cells.** It reflects the 72 h MTT values and compares single dose treatment with combination doses and determines the most effective interaction and can be interpreted by combination index (CI) values. CI values range from 0 to 1. The lower the CI values, the more effective is the interaction and the stronger the synergism and vice versa. (PDF 139 kb)
Additional file 2: **Is the report generated after using CompuSyn software to calculate the synergism in MDA-MB-157 breast cancer cells.** It reflects the 72 h MTT values and compares single dose treatment with combination doses and determines the most effective interaction and can be interpreted by combination index (CI) values. CI values range from 0 to 1. The lower the CI values, the more effective is the interaction and the stronger the synergism and vice versa. (PDF 110 kb)

## Abbreviations

CI: Combination index
TNBC: Triple-negative breast cancers
SIRT1: Sirtuin (silence mating type information regulation 2 homolog)
HDAC: Histone deacetylases enzyme
DDR: DNA damage response
γ-H2AX: Gamma-H2A histone family member X
IF: Immunofluorescence
DNMT: DNA methyltransferase enzyme
hTERT: Human telomerase reverse transcriptase
TRAP: Telomeric repeat amplification protocol
