# Supplementary material for: Epigenetic-based combinatorial resveratrol and pterostilbene alters DNA damage response by affecting SIRT1 and DNMT enzyme expression, including SIRT1-dependent γ-H2AX and telomerase regulation in triple-negative breast cancer
Source: BMC Cancer. 2015 Oct 12;15:672. doi: 10.1186/s12885-015-1693-z (PMC4603342; doi:10.1186/s12885-015-1693-z)
Supplement: Additional file 1: — Is the report generated after using CompuSyn software to calculate the synergism in HCC1806 breast cancer cells. It reflects the 72 h MTT values and compares single dose treatment with combination doses and determines the most effective interaction and can be interpreted by combination index (CI) values. CI values range from 0 to 1. The lower the CI values, the more effective is the interaction and the stronger the synergism and vice versa. (PDF 139 kb) [file 12885_2015_1693_MOESM1_ESM.pdf]

CompuSyn Report

Experiment Name: HCC1806 72 h
Date:
File Name: C:\Users\Rishabh\Desktop\CI calculation\HCC1806 three day.cse
Description

Drug: Resveratrol (Res) [micromolar]
Drug: Pterostilbene (Ptero) [micromolar]
Drug Combo: Res/Ptero (R/P) (Res+Ptero)

Data for Drug: Res [micromolar]

Dose	Effect
5.0	0.87
10.0	0.87
15.0	0.701

3 data points entered.

X-int: 1.74918
Y-int: 1.49019 +/- 0.66533
m: -0.8519 +/- 0.68002
Dm: 56.1280
r: -0.7816

Data for Drug: Ptero [micromolar]

Dose	Effect
5.0	0.825
10.0	0.732
15.0	0.634

3 data points entered.

X-int: 1.45889
Y-int: 1.31004 +/- 0.08633
m: -0.8980 +/- 0.08824
Dm: 28.7665
r: -0.9952

Data for Non-Constant Combo: R/P (Res+Ptero)

Dose Res	Dose Ptero	Effect
5.0	5.0	0.848
5.0	10.0	0.63
5.0	15.0	0.688
10.0	5.0	0.651
10.0	10.0	0.637

10.0	15.0	0.439
15.0	5.0	0.485
15.0	10.0	0.509
15.0	15.0	0.409

9 data points entered.

Dose-Effect Curve

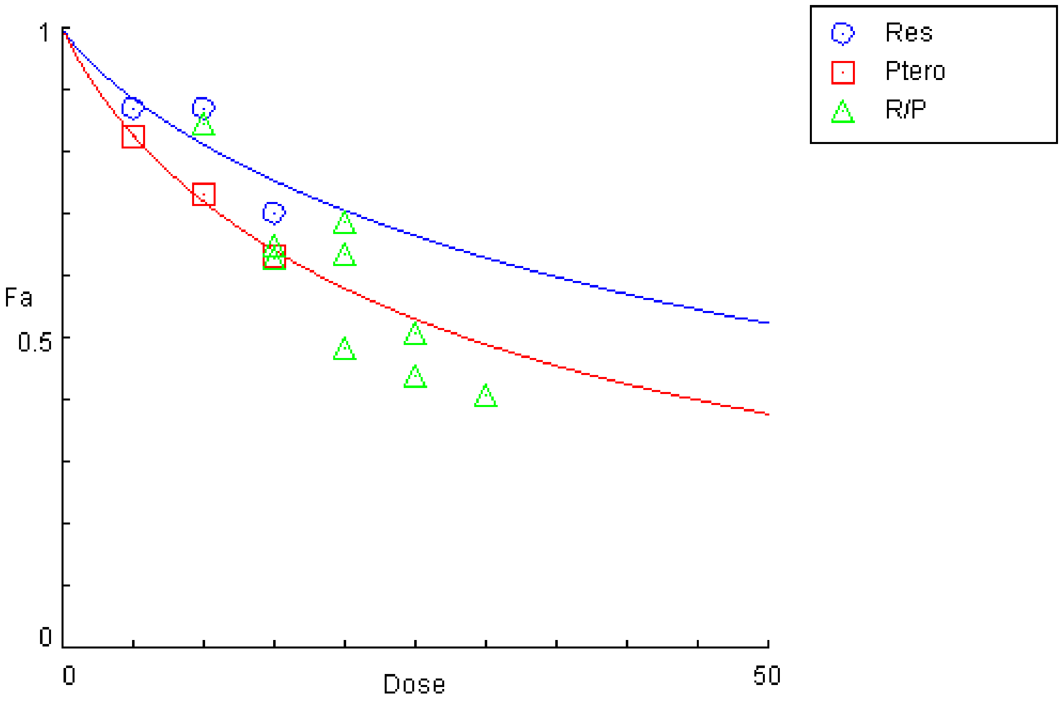

Median-Effect Plot

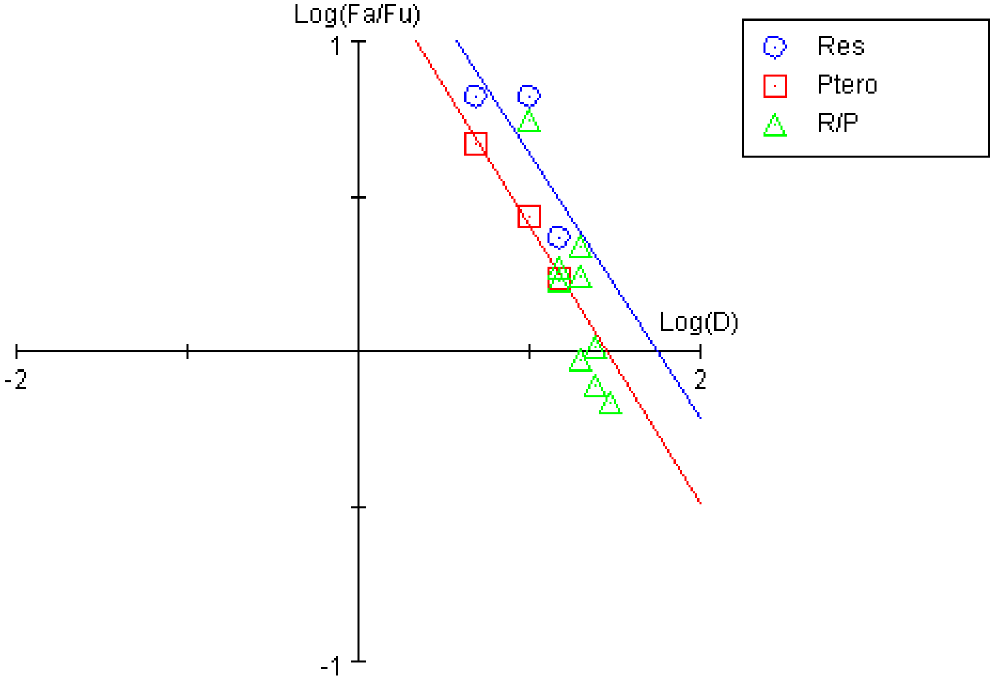

CI Data for Non-Constant Combo: R/P (Res+Ptero)

Dose Res	Dose Ptero	Effect	CI
5.0	5.0	0.848	1.84887
5.0	10.0	0.63	0.79518
5.0	15.0	0.688	1.48331
10.0	5.0	0.651	0.71838
10.0	10.0	0.637	0.99502
10.0	15.0	0.439	0.53043
15.0	5.0	0.485	0.41164
15.0	10.0	0.509	0.64063
15.0	15.0	0.409	0.51957

Combination Index Plot

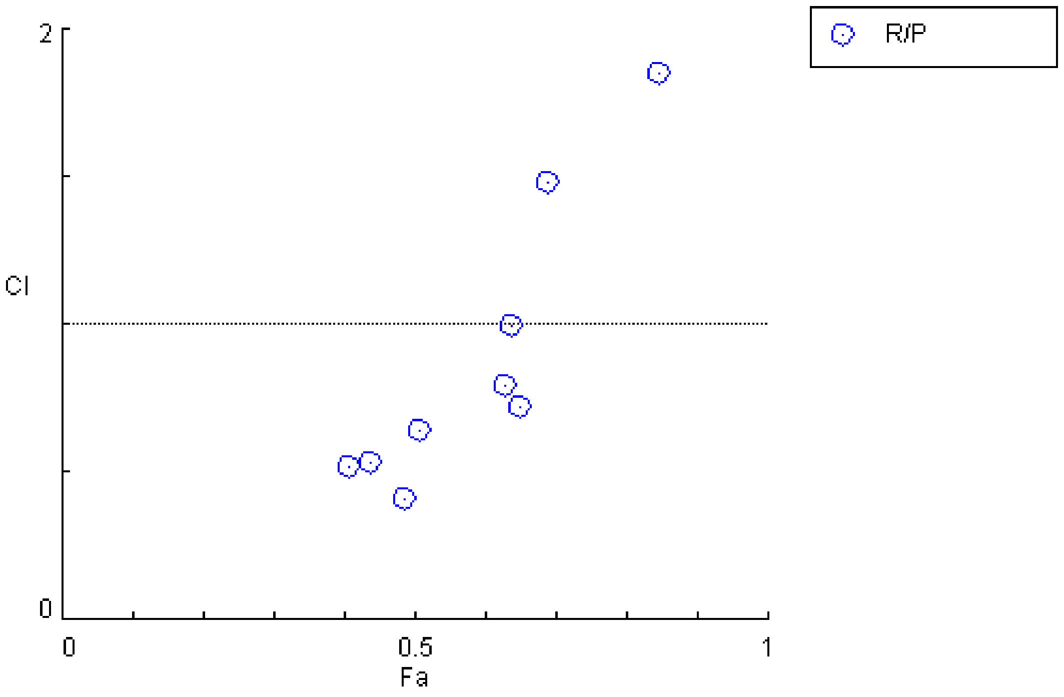

DRI Data for Non-Constant Combo: R/P (Res+Ptero)

Fa	Dose Res	Dose Ptero	DRI Res	DRI Ptero
0.848	7.46240	4.24145	1.49248	0.84829
0.63	30.0517	15.9033	6.01034	1.59033
0.688	22.1849	11.9243	4.43698	0.79496
0.651	27.0002	14.3671	2.70002	2.87342
0.637	29.0068	15.3782	2.90068	1.53782
0.439	74.8492	37.7995	7.48492	2.51997
0.485	60.2248	30.7549	4.01498	6.15099
0.509	53.8054	27.6360	3.58702	2.76360
0.409	86.4625	43.3426	5.76416	2.88951

DRI Plot for Non-Constant Combo: R/P (Res+Ptero)

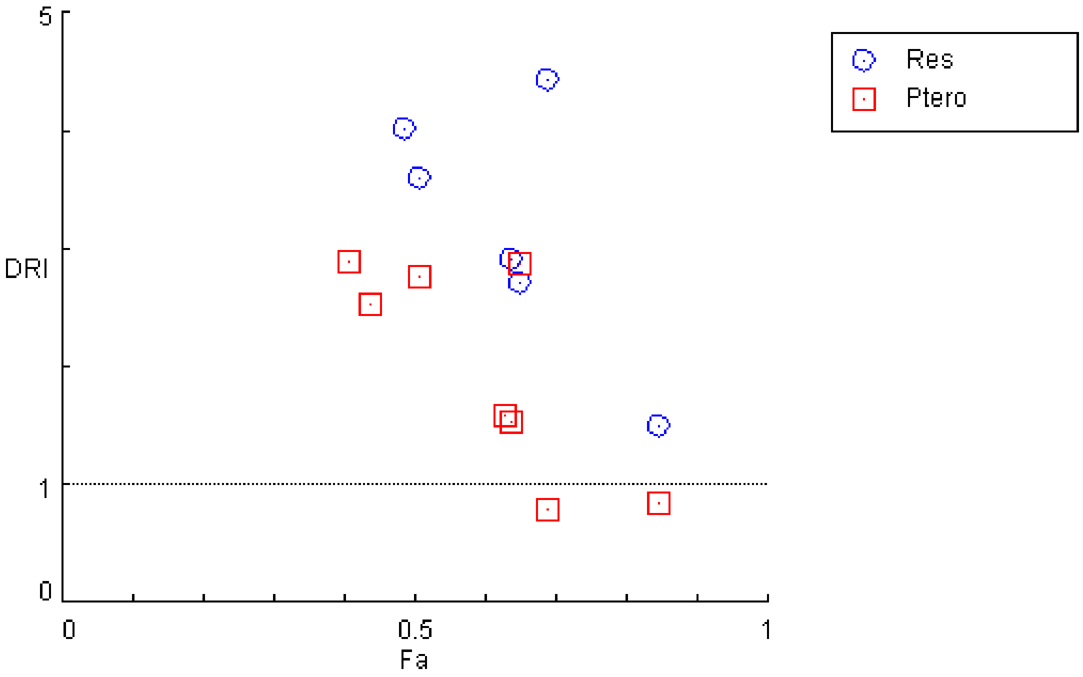

Normalized Isobologram for Combo: R/P (Res+Ptero)

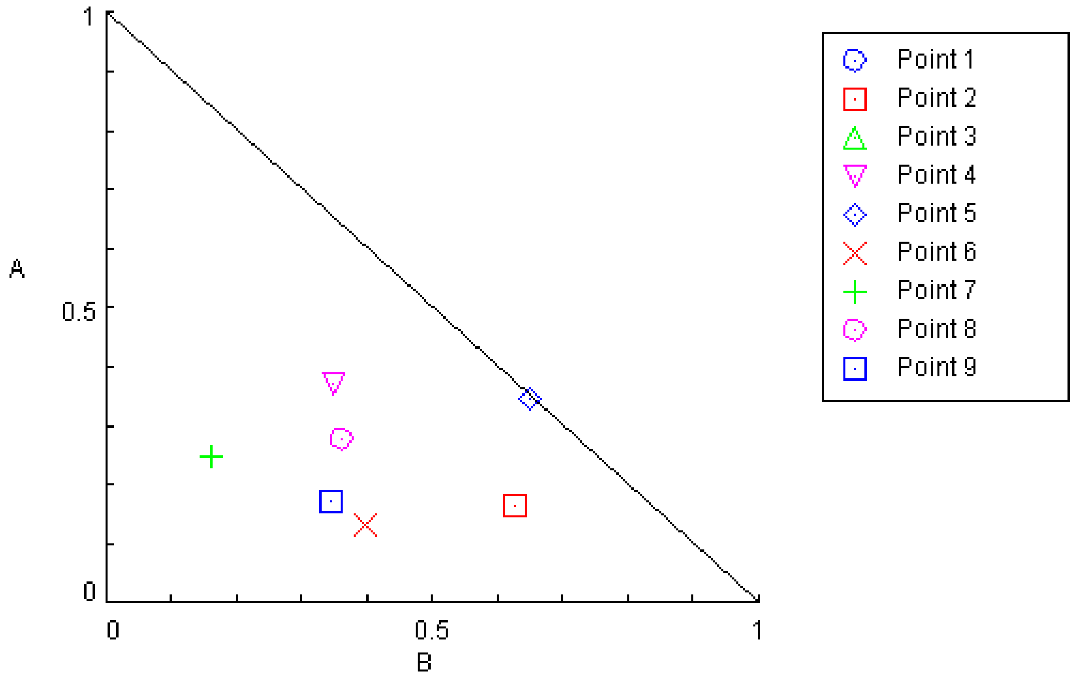

Summary Table

Experiment Name: HCC1806 72 h

Date:

File Name: C:\Users\Rishabh\Desktop\CI calculation\HCC1806 three day.cse

Description

Drug: Resveratrol (Res) [micromolar]
Drug: Pterostilbene (Ptero) [micromolar]
Drug Combo: Res/Ptero (R/P) (Res+Ptero)

Drug/Combo	Dm	m	r
Res	56.1280	-0.8519	-0.7816
Ptero	28.7665	-0.8980	-0.9952

CI values at:

Combo ED50 ED75 ED90 ED95

Data for Fa = 0.5

Drug/Combo	CI value	Dose Res	Dose Ptero
Res		56.1280	
Ptero			28.7665

Data for Fa = 0.75

Drug/Combo	CI value	Dose Res	Dose Ptero
Res		15.4574	
Ptero			8.46364

Data for Fa = 0.9

Drug/Combo	CI value	Dose Res	Dose Ptero
Res		4.25689	
Ptero			2.49016

Data for Fa = 0.95

Drug/Combo	CI value	Dose Res	Dose Ptero
Res		1.77085	
Ptero			1.08354

Data for Fa = 0.97

Drug/Combo	CI value	Dose Res	Dose Ptero
Res		0.94876	
Ptero			0.59940
