# Supplementary material for: Epigenetic-based combinatorial resveratrol and pterostilbene alters DNA damage response by affecting SIRT1 and DNMT enzyme expression, including SIRT1-dependent γ-H2AX and telomerase regulation in triple-negative breast cancer
Source: BMC Cancer. 2015 Oct 12;15:672. doi: 10.1186/s12885-015-1693-z (PMC4603342; doi:10.1186/s12885-015-1693-z)
Supplement: Additional file 2: — Is the report generated after using CompuSyn software to calculate the synergism in MDA-MB-157 breast cancer cells. It reflects the 72 h MTT values and compares single dose treatment with combination doses and determines the most effective interaction and can be interpreted by combination index (CI) values. CI values range from 0 to 1. The lower the CI values, the more effective is the interaction and the stronger the synergism and vice versa. (PDF 110 kb) [file 12885_2015_1693_MOESM2_ESM.pdf]

CompuSyn Report

Experiment Name: MDA-MB-157 72 h
Date:
File Name: C:\Users\Rishabh\Desktop\CI calculation\.cse
Description

Drug: Resveratrol (Res) [micromolar]
Drug: Pterostilbene (Ptero) [micromolar]
Drug Combo: Res/Ptero (R/P) (Res+Ptero)

Data for Drug: Res [micromolar]

Dose	Effect
5.0	0.975
10.0	0.951
15.0	0.886

3 data points entered.

X-int: 1.84413
Y-int: 2.61601 +/- 0.32156
m: -1.4186 +/- 0.32867
Dm: 69.8435
r: -0.9742

Data for Drug: Ptero [micromolar]

Dose	Effect
5.0	0.925
10.0	0.778
15.0	0.754

3 data points entered.

X-int: 1.49171
Y-int: 1.97843 +/- 0.38188
m: -1.3263 +/- 0.39032
Dm: 31.0248
r: -0.9593

Data for Non-Constant Combo: R/P (Res+Ptero)

Dose Res	Dose Ptero	Effect
5.0	5.0	0.735
5.0	10.0	0.699
5.0	15.0	0.702
10.0	5.0	0.709
10.0	10.0	0.677
10.0	15.0	0.684
15.0	5.0	0.568
15.0	10.0	0.553

15.0	15.0	0.477

9 data points entered.

Dose-Effect Curve

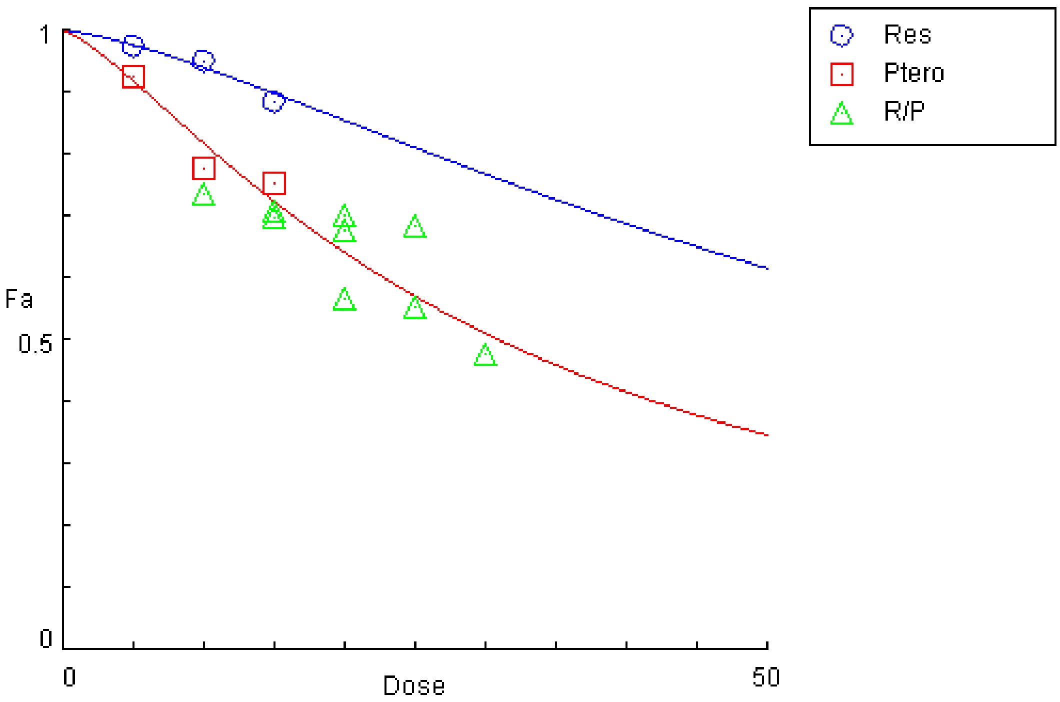

Median-Effect Plot

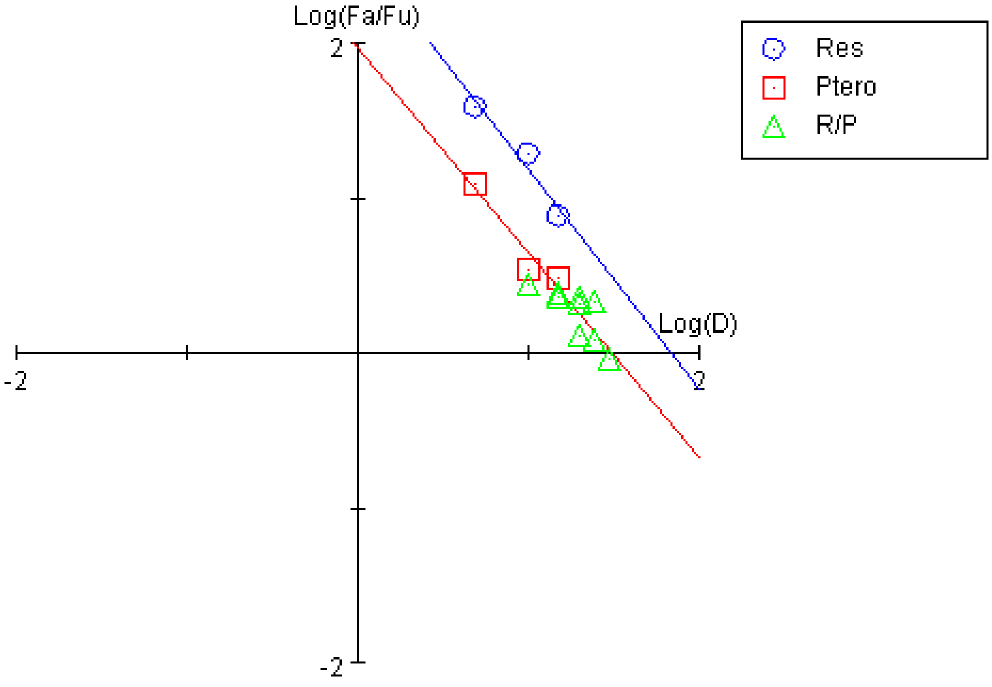

CI Data for Non-Constant Combo: R/P (Res+Ptero)

**Dose Res**	**Dose Ptero**	**Effect**	**CI**
5.0	5.0	0.735	0.49473
5.0	10.0	0.699	0.73805
5.0	15.0	0.702	1.05345
10.0	5.0	0.709	0.58364

10.0	10.0	0.677	0.80436
10.0	15.0	0.684	1.11223
15.0	5.0	0.568	0.45857
15.0	10.0	0.553	0.62794
15.0	15.0	0.477	0.65233

Combination Index Plot

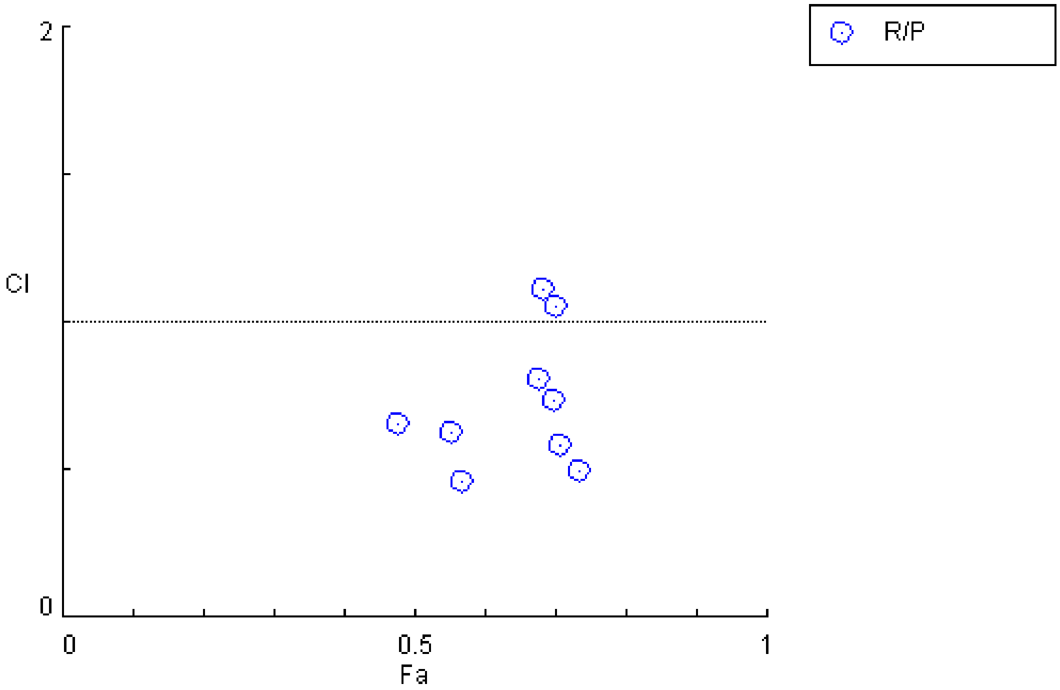

DRI Data for Non-Constant Combo: R/P (Res+Ptero)

Fa	Dose Res	Dose Ptero	DRI Res	DRI Ptero
0.735	34.0259	14.3768	6.80518	2.87536
0.699	38.5640	16.4368	7.71279	1.64368
0.702	38.1772	16.2605	7.63544	1.08404
0.709	37.2811	15.8527	3.72811	3.17053
0.677	41.4542	17.7578	4.14542	1.77578
0.684	40.5239	17.3319	4.05239	1.15546
0.568	57.5882	25.2398	3.83922	5.04797
0.553	60.1142	26.4257	4.00761	2.64257
0.477	74.5266	33.2549	4.96844	2.21699

DRI Plot for Non-Constant Combo: R/P (Res+Ptero)

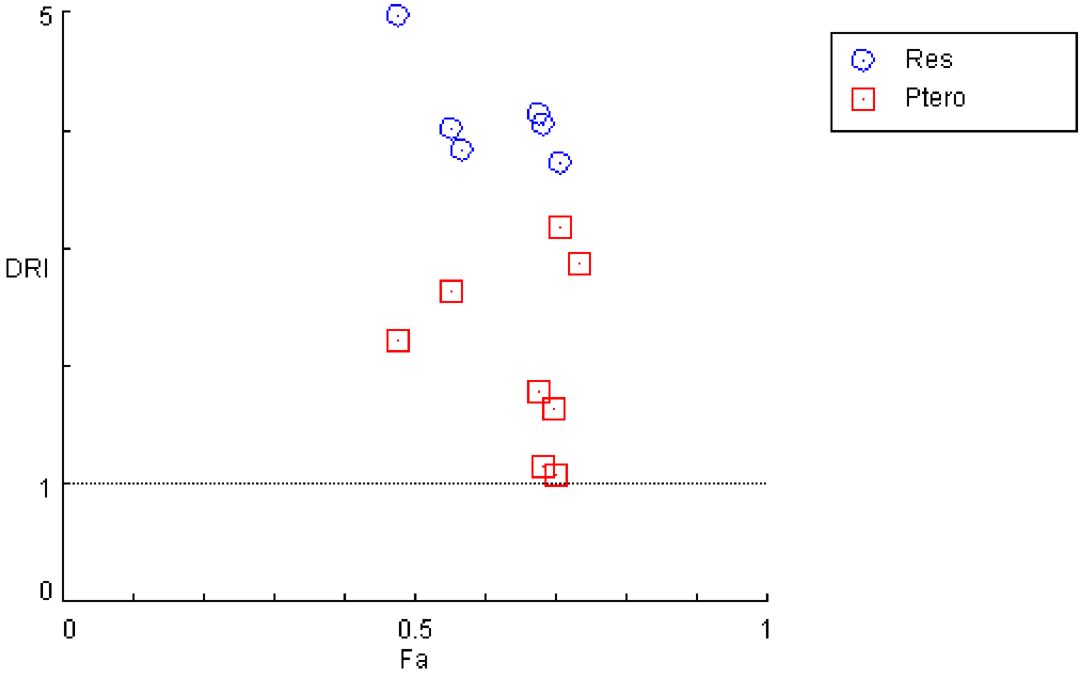

Normalized Isobologram for Combo: R/P (Res+Ptero)

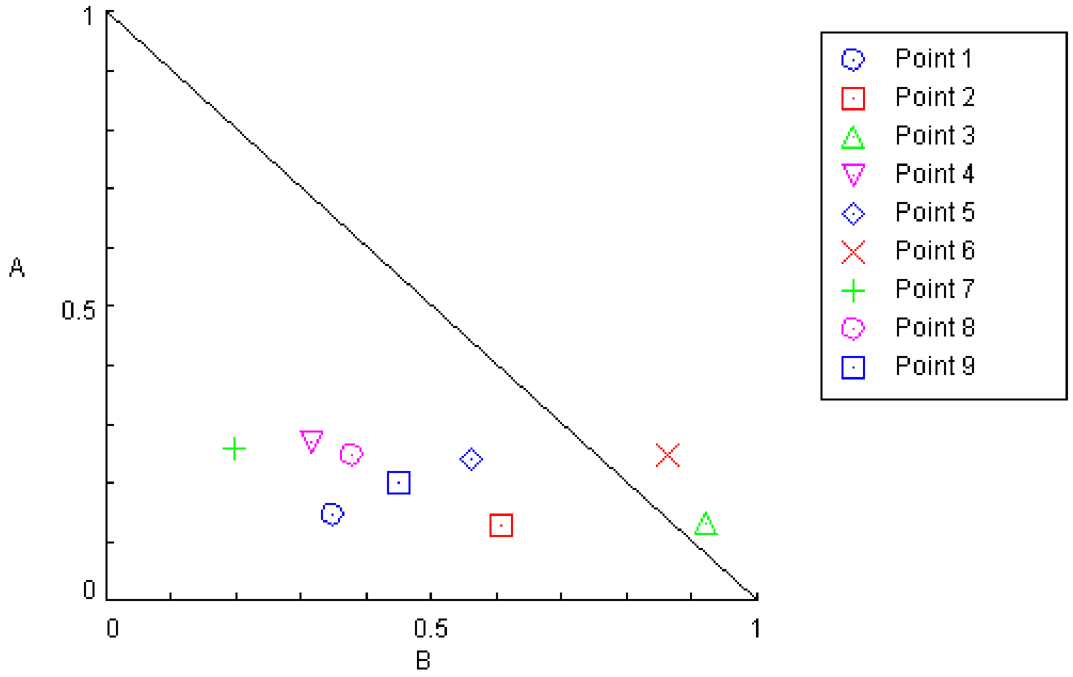

Summary Table

Experiment Name: MDA-MB-157 72 h

Date:

File Name: C:\Users\Rishabh\Desktop\CI calculation\.cse

Description

Drug: Resveratrol (Res) [micromolar]

Drug: Pterostilbene (Ptero) [micromolar]

Drug Combo: Res/Ptero (R/P) (Res+Ptero)

Drug/Combo	Dm	m	r
Res	69.8435	-1.4186	-0.9742
Ptero	31.0248	-1.3263	-0.9593

CI values at:

Combo	ED50	ED75	ED90	ED95

Data for Fa = 0.5

Drug/Combo	CI value	Dose Res	Dose Ptero
Res		69.8435	
Ptero			31.0248

Data for Fa = 0.75

Drug/Combo	CI value	Dose Res	Dose Ptero
Res		32.1948	
Ptero			13.5508

Data for Fa = 0.9

Drug/Combo	CI value	Dose Res	Dose Ptero
Res		14.8404	
Ptero			5.91867

Data for Fa = 0.95

Drug/Combo	CI value	Dose Res	Dose Ptero
Res		8.76364	
Ptero			3.36936

Data for Fa = 0.97

Drug/Combo	CI value	Dose Res	Dose Ptero
Res		6.02444	
Ptero			2.25660
